# IPO: a tool for automated optimization of XCMS parameters

**DOI:** 10.1186/s12859-015-0562-8

**Published:** 2015-04-16

**Authors:** Gunnar Libiseller, Michaela Dvorzak, Ulrike Kleb, Edgar Gander, Tobias Eisenberg, Frank Madeo, Steffen Neumann, Gert Trausinger, Frank Sinner, Thomas Pieber, Christoph Magnes

**Affiliations:** 1 Joanneum Research Forschungsgesellschaft m.b.H., HEALTH, Institute for Biomedicine and Health Sciences, Graz, Austria; 2 Joanneum Research Forschungsgesellschaft m.b.H., POLICIES, Institute for Economic and Innovation Research, Graz, Austria; 3 Institute of Molecular Biosciences, NAWI Graz, University of Graz, 8010 Graz, Austria; 4 BioTechMed Graz, 8010 Graz, Austria; 5 Department of Stress- and Developmental Biology, Leibniz Institute of Plant Biochemistry, Halle, Germany; 6 Department of Internal Medicine, Medical University of Graz, Graz, Austria

**Keywords:** Metabolomics, XCMS, Parameter optimization, Design of experiments, Isotopologue

## Abstract

**Background:**

Untargeted metabolomics generates a huge amount of data. Software packages for automated data processing are crucial to successfully process these data. A variety of such software packages exist, but the outcome of data processing strongly depends on algorithm parameter settings. If they are not carefully chosen, suboptimal parameter settings can easily lead to biased results. Therefore, parameter settings also require optimization. Several parameter optimization approaches have already been proposed, but a software package for parameter optimization which is free of intricate experimental labeling steps, fast and widely applicable is still missing.

**Results:**

We implemented the software package IPO (‘Isotopologue Parameter Optimization’) which is fast and free of labeling steps, and applicable to data from different kinds of samples and data from different methods of liquid chromatography - high resolution mass spectrometry and data from different instruments.

IPO optimizes XCMS peak picking parameters by using natural, stable ^13^C isotopic peaks to calculate a peak picking score. Retention time correction is optimized by minimizing relative retention time differences within peak groups. Grouping parameters are optimized by maximizing the number of peak groups that show one peak from each injection of a pooled sample. The different parameter settings are achieved by design of experiments, and the resulting scores are evaluated using response surface models. IPO was tested on three different data sets, each consisting of a training set and test set. IPO resulted in an increase of reliable groups (146% - 361%), a decrease of non-reliable groups (3% - 8%) and a decrease of the retention time deviation to one third.

**Conclusions:**

IPO was successfully applied to data derived from liquid chromatography coupled to high resolution mass spectrometry from three studies with different sample types and different chromatographic methods and devices. We were also able to show the potential of IPO to increase the reliability of metabolomics data.

The source code is implemented in R, tested on Linux and Windows and it is freely available for download at https://github.com/glibiseller/IPO. The training sets and test sets can be downloaded from https://health.joanneum.at/IPO.

**Electronic supplementary material:**

The online version of this article (doi:10.1186/s12859-015-0562-8) contains supplementary material, which is available to authorized users.

## Background

Untargeted metabolomics screens biological samples with the aim to reveal new compounds and to understand biological mechanisms. Untargeted metabolomics by using liquid chromatography (LC) generates a huge amount of data when coupled to mass spectrometry (MS). Software packages for automated data processing are needed to successfully process large data sets. Recently, a tool MetExtract has been presented which uses carbon labeling with stable isotopes to find reliable peaks [1,2]. This tool increases the selectivity of compounds with biological origin, performs feature reduction and assesses molecular structures of measured substances. Disadvantages of MetExtract are the time and the cost intensive labeling step and its feasibility which is limited to samples that can be labeled.

A number of software packages for processing LC-MS data have already been developed for data sets of samples that do not rely on labeling [3-12]. They provide methods for peak detection, peak picking, retention time correction and grouping and offer a variety of adjustable parameters to provide reasonable results. But even though these parameters are intended to optimize the results, wrong parameter selection can lead to distorted outcomes. Parameter optimization is necessary to counter wrong selection. Up to now, several parameter optimization approaches have been proposed to increase the reliability of the results [13-15].

One parameter optimization approach uses design of experiments (DoE) [13]. A designed experiment is a series of tests in which specific modifications are made to the input variables of a process. DoE aims to optimize the response to modifications or to either explain changes of the response variable. . For metabolomics data a dilution series of a pooled sample is measured and a reliability index for each experiment of the DoE is calculated. This reliability index is based on the assumption that peaks which correlate with the dilution series are reliable ones, and those which do not correlate are unreliable peaks. The DoE optimization approach provides quality evaluation of the resulting optimization, but is very time intensive. To accelerate the DoE optimization approach, Zheng, H et al. [14] refined the workflow by first applying a screening step prior to the optimization. Screening steps are usually performed in the first stage of an optimization process with the purpose of identifying the parameters that have large effects on the target variable. For the screening step Zhen et al. used a Plackett-Burman design. Such a fractional factorial design defines only two levels for each parameter and thus requires relatively few experiments. Two levels stand for two different tested values for each parameter. Second, only parameters with a significant positive influence on the target value are optimized and thus the overall optimization time is considerably decreases. However, potential important parameters may be lost because they may fall into a range where they do not significantly influence the target value and hence they may not be further optimized. A software package for parameter optimization which is even faster, widely applicable and free of intricate labeling steps is still missing.

To close this gap we implemented the R-package IPO (‘Isotopologue Parameter Optimization’) that exploits natural, stable ^13^C isotope peaks which are ubiquitously present in biological samples. The use of these ^13^C isotope peaks makes all labeling steps expendable and leads to the calculation of a target value to assess the optimization quality. IPO increases the reliability of peak picking, retention time correction and grouping results and starts the optimization process for the parameters to be optimized at the respective default settings of the XCMS methods and is thus also well suited for inexperienced XCMS users.

## Implementation

**Figure 1:**
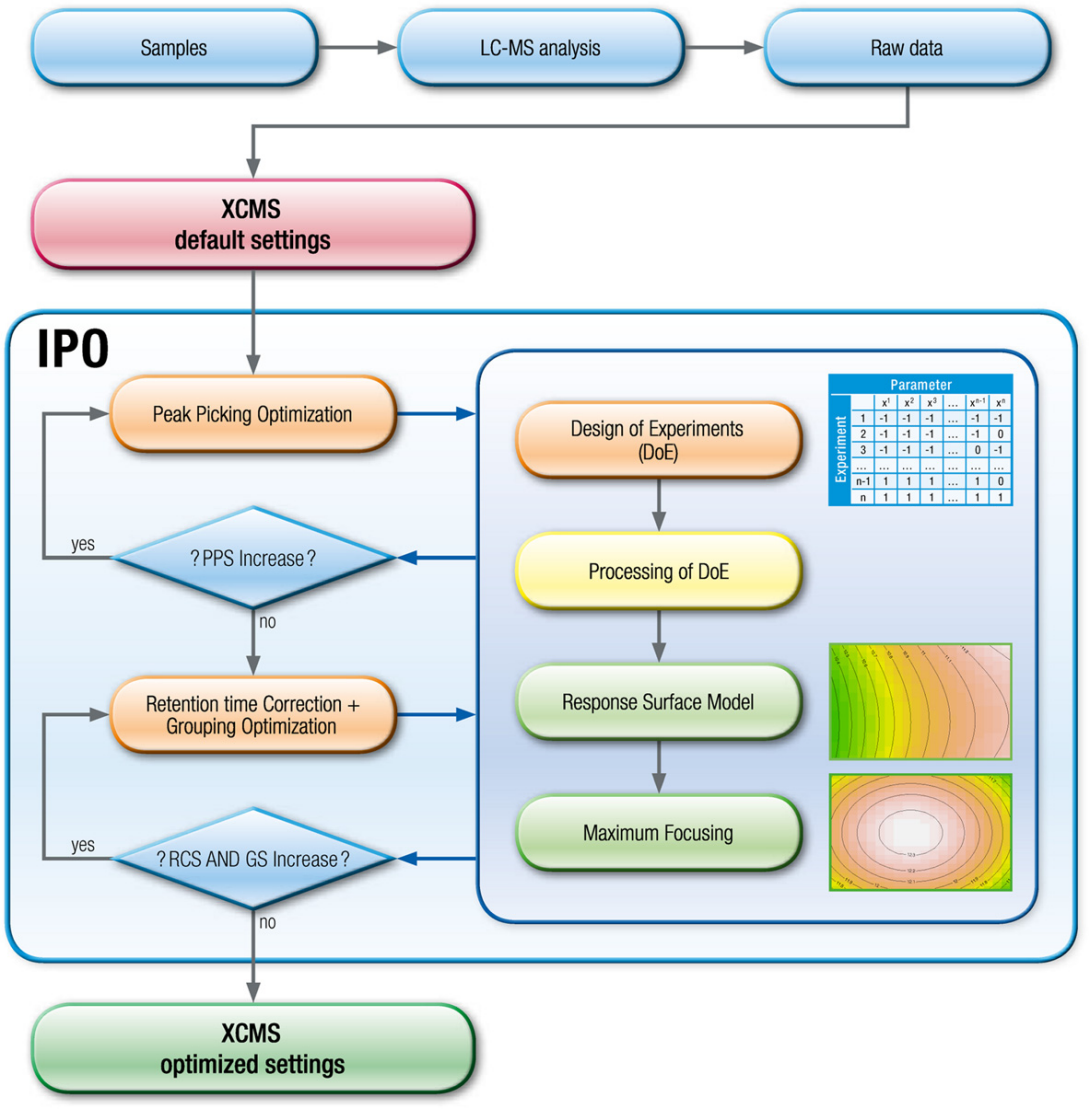
Workflow for the optimization of XCMS parameter. A pooled sample is measured sequentially within the studies. The LC-MS data of the pooled sample are then used for optimization. The DoEs are created by using Box-Behnken designs. The individual experiments of the design are calculated in parallel. Peaks are classified as reliable peaks (RP) when they are part of an isotopologue. These RPs serve as basis for the calculation of the Peak Picking Score (PPS). Two additional scores are introduced for retention time correction and grouping. To improve the quality of retention time correction, the relative retention time deviations within the peak groups are minimized which leads to the Retention time Correction Score (RCS). So called ‘reliable groups’ and ‘non-reliable groups’ are defined to assess grouping. The ratio of the squared number of ‘reliable groups’ to ‘non-reliable groups’, the Grouping Score (GS), is maximized within the optimization process. The resulting scores are evaluated by using response-surface-models. The combination of parameters that yields the best score is used as new center for the next DoE. The optimization process continues as long as the respective scores are increasing.

We developed the R-package IPO to optimize parameters of the open-source package XCMS [3,4]. The process for the parameter optimization by IPO is described in the following subsections (Figure 1).

### XCMS parameters

Metabolomics data processing requires peak picking followed by retention time correction and grouping. Multiple methods for each of these steps are provided by XCMS. IPO supports two peak picking, one retention time correction and one grouping method, and can be extended to cover other methods in the future. Various parameters of these methods are optimized by default (Table 1); all other quantitative parameters are optimized only if defined by the user.

**Table 1:** **XCMS methods and their respective parameters optimized by IPO**

**XCMS method**	**Parameters**
xcmsSet(method = ‘centWave’)	min peakwidth, max peakwidth, ppm, mzdiff
xcmsSet(method = ‘matchedFilter’)	fwhm, step, steps, snthresh, mzdiff
retcor(method = ‘obiwarp’)	profStep, gapInit, gapExtend
group(method = ‘density’)	bw, mzwid, minfrac

The first ‘xcmsSet’-method ‘centWave’ [16] deals with **peak picking**. This is the method of choice for processing centroided data acquired with liquid chromatography (LC) coupled to high resolution mass spectrometry (HRMS). First, ‘centWave’ identifies regions of interest (ROIs). ROIs are created by combining consecutive centroids within a tolerated m/z deviation, defined by the parameter ‘ppm’. Chromatographic peaks are identified within the ROIs using wavelets. The peak width parameters (‘min peakwidth‘ and ‘max peakwidth‘) describe the range of the expected peak widths and determine the scales of the wavelets. The minimum difference of m/z for peaks with overlapping retention times is given by ‘mzdiff’.

The second ‘xcmsSet’-method ‘matchedFilter’ [3] also deals with **peak picking**, but it has particularly been developed for low resolution data. In our study, we only optimized high resolution data and therefore we present no example for a parameter optimization with ‘matchedFilter’. Nevertheless, IPO also supports this method. The LC-MS data is cut into m/z slices. The widths of these slices are defined by the parameter ‘step’ and multiple slices can be combined to avoid issues at the boundaries. The parameter ‘steps’ defines the number of adjacent slices to be combined. Matched filtration is used to filter these slices with a second-derivative Gaussian model peak shape. This Gaussian model peak shape is defined by the parameter ‘fwhm’. A signal to noise ratio to filter noisy peaks is determined by the ‘snthresh’ parameter.

The ‘obiwarp’ method (Table 1) is responsible for the **retention time correction** [17]. The ‘center’ parameter indicates the sample which serves as reference sample for retention time correction. If not otherwise specified by the user, XCMS uses the sample with the highest number of peaks as ‘center’ sample whereas IPO chooses the one with the highest average intensity. First, profiles are generated from the raw data. The parameter ‘profStep’ defines the widths of these profiles in the m/z dimension. Then, the profiles are compared to each other and a similarity matrix is calculated. Similarity scores are added to recursively generate an optimal path. Off-diagonal transitions are penalized. The parameters ‘gapInit’ and ‘gapExtend’ define penalties for gap openings and gap enlargements, respectively.

The XCMS method ‘density’ is a method for the **grouping** step. Grouping is the process of combining peaks from different samples with similar masses and retention times to peak groups. The parameter ‘bw’ is used to define a certain retention time range to find peak groups. ‘mzwid’ describes the allowed variation in the m/z dimension. The default value for ‘mzwid’ is 0.25 which is too high for high resolution data and this value was therefore set to 0.025. A valid feature must have a minimum fraction of samples within at least one sample group. This fraction is defined by the parameter ‘minfrac’.

### Optimization procedure

In general, peak picking is done for each individual data file but for retention time correction and grouping multiple data files are necessary. The optimization procedure splits the parameters by applying a semi sequential approach. Peak picking parameters are optimized first and the retention time correction and grouping parameters are simultaneously optimized afterwards. Grouping results in peak groups by combining peaks with similar masses and retention times from different LC-MS runs. Simultaneous optimization of retention time correction and grouping is necessary because grouping is required for the assessment of the retention time correction step, which in turn can improve the grouping. This semi-sequential approach additionally decreases the overall computing time. The different levels for the XCMS parameters are determined by a design of experiments approach [18]. Box-Behnken designs (BBD) serve as basis to generate the DoEs. BBD is a three level incomplete factorial design for fitting a second order response surface model. Three levels denote that for each parameter three different evenly spaced values are tested. The two outer values define a range, the middle value a center point. In contrast to a full factorial design, BBD does not test all factorial combinations, making it highly efficient [19]. For the default levels used by IPO in the first DoE see Additional file 1. To evaluate the result of the DoE, one score for peak picking and one score for retention time correction and grouping is used.

### Peak picking

IPO supports the peak picking methods ‘centWave’ and ‘matchedFilter’. By using isotopic peaks it is possible to assess the reliability of peak picking by calculating a peak picking score (*PPS*):

1 $$ PPS=\frac{R{P}^2}{` all\  peaks' - LIP} $$

The PPS is defined as the ratio of reliable peaks (*RP*s) to the number of all peaks (*all peaks*), diminished by the number of ‘low intensity peaks’ (*LIP*). RP is weighted by the exponential factor 2. Therefore, if the RP value and the number of all peaks increase by the same amount, the PPS increases. This creates an optimization force towards an increased recall of reliable peaks. The exponent value of 2 is an empirical one. The sensitivity for RPs could be enhanced by further increasing this exponent, but then noise would also rise. RPs are defined as peaks that belong to an isotopologue. IPO identifies isotopologues consisting of ^13^C isotope peaks, which are defined by three criteria. Only peaks that meet all these three criteria are considered isotopic peaks. The tolerable ranges of these criteria are calculated relative to the respective ^12^C peak. The first criterion states that the mass of the isotope peak has to be within a certain mass range. Second, the isotopic peak must elute at the same time as the parent peak. To restrict peaks on the time axis, a relative retention time window is specified. As a third criterion, the intensities of isotopic peak candidates have to be within a certain intensity window. Therefore, the maximum number of possible carbon atoms (*maxC*) for a specific mass-to-charge ratio presuming a hydrocarbon chain is estimated as follows:

2 $$ maxC= floor\left(\frac{m/z-2*CH3}{CH2}\right)+2 $$

*m/z* is the mass-to-charge ratio of a peak. *CH2* is the mass of a molecule consisting of one carbon atom and two hydrogen atoms and *CH3* depicts the exact mass of a molecule consisting of one carbon and three hydrogen atoms respectively. First m/z is reduced by 2*CH3 which represent the ends of a hydrocarbon chain. Then, the difference is divided by CH2 which is exemplary for the hydrocarbon bonds within the chain. The function *floor* is used on the result to cut of fractional digits. The previously subtracted 2*CH3 from the ends of the hydrocarbon chain is compensated by *+ 2* to calculate maxC. Then, intensities of the isotope peaks with one carbon atom and with maxC carbon atoms are estimated by multiplying one and maxC with the natural abundance of ^13^C isotopes and the ^12^C peak’s intensity. Consequently an intensity window is defined. ‘all peaks’ includes reliable as well as unreliable peaks. We consider the fact that reliable peaks may exist whose isotope peak concentrations are too low to measure, and would falsely be classified as unreliable ones. To counter this, all peak intensities are arranged in descending order and the average of the lower three percent of the peak intensities is calculated as cut-off value. This cut-off value is used to estimate the sensitivity of the LC-MS system.

For each peak, except for the RPs, the maximum amount of possible carbon atoms is estimated and this amount is then multiplied with the natural ^13^C isotopic abundance, IA. If the intensity of the peak lies below the cut-off value when multiplied with IA, the peak is neither reliable nor unreliable and is defined as *LIP*.

### Retention time correction and grouping

Run-to-run retention time changes have to be corrected. To assess the quality of the retention time correction for one peak group, a group retention time shift (*GRTS*) is calculated as follows:

3 $$ GRTS(x)=\frac{{\displaystyle {\sum}_{n=1}^k}\left|\left( median(x)-{x}_n\right)\right|}{k} $$

*x* are the retention times of all peaks within one group, *k* is the number of these retention times and *n* is an index pointing at the retention time of one individual peak in the peak group. *median(x)* calculates the median value of the retention times for all peaks in one group. For every *x* the difference to the median retention time is calculated. The average of all these differences is defined as GRTS. The average of all GRTS values yields the average retention time shifts (*ARTS*):

4 $$ ARTS=\frac{1}{k}* sum(GRTS) $$

The number of all GRTSs is defined by *k* and the function *sum* calculates the sum of these GRTSs. Decreasing the ARTS improves the result. To create a usable optimization value for maximization, the inverse of ARTS is used to define a retention time correction score (*RCS*):

5 $$ RCS=\frac{1}{ARTS} $$

The grouping score (*GS*) is based on the classification of peak groups into ‘reliable’ and ‘non-reliable’ ones. ‘*Reliable groups*’ are assumed to show exactly one peak from each injection of a pooled sample. All groups that do not obey this assumption are classified as ‘*non-reliable groups*’. The absence of a peak within a group can occur due to retention time shifts or due to too low concentrations. GS is calculated as follows:

6 $$ GS=\frac{{` reliable\  groups'}^2}{`non- reliable\  groups'} $$

The squared number of ‘reliable groups’ divided by the number of ‘non-reliable groups’ is defined as GS. Calculation of the retention time correction and grouping target value (RGTV) is done by the following formula:

7 $$ RGTV= norm(RCS)+ norm(GS) $$

To balance the impact of RCS and GS on RGTV, the function ‘*norm*’ is used on RCS as well as on GS. Here, norm is a unity-based normalization used on all RCS values of the experiments of one DoE to scale these values between 0 and 1. The same is done for GS. The normalized values of the same experiments are added giving one RGTV for each experiment of a DoE.

### DoE evaluation and adjustment

After the respective scores for each experiment of the DoE have been calculated, response surface models are estimated and applied to evaluate the quality of peak picking, retention time correction and grouping. In a ‘maximum focusing step’ the combination of parameters which leads to the best respective score is found and used as the new center point for the next DoE. Additionally, in this step, parameter ranges are adjusted according to the following procedure: If the maximum of a parameter shows the same value on the upper and on the lower bound of the parameter range, the range is increased by 20% (zooming out). If the maximum of a parameter has already been located in the middle of the parameter range, with a deviation of less than 25% from the center point, the tool ‘zooms in’ by narrowing the parameter range by 10% at each bound. The adjusted DoE is recalculated. As long as the respective scores are increasing, this process is continued.

## Results and discussion

IPO was applied to untargeted metabolomics data from three different studies that were using different chromatographic devices and methods [20-22]. The sample data originated from human serum, animal tissue (mouse muscle, lung, heart) and yeast samples. All data were high resolution data deriving from LC-HRMS instruments. The three studies used different chromatographic methods that provide data differing in number, shape and quality of the resulting peaks. See Additional file 2 for the characteristics of the data sets. The parameter settings were optimized on training sets and these optimized settings were used on the training and an independent test set. The test set gives an unbiased view of the improvement that can be expected from the approach. The results of the test sets with regard to the parameter optimization steps are presented in Table 2. All response surface models generated during the optimization process of the three data sets are presented in Additional file 3.

**Table 2:** **Results of the example data sets**

	**Metabolite fingerprinting**		**Lipidomics**		**Central carbon metabolism**	
pooled sample injections						
training set:	12		4		6	
test set:	11		4		6	
DoEs peakpicking	4		3		2	
DoEs retcor + grouping	5		5		4	
time for peakpicking optimization	3.8 h		1.5 h		0.9 h	
time for retcor + grouping optimization	0.8 h		0.7 h		0.6 h	
overall time	4.6 h		2.2 h		1.5 h	
	default	optimized	default	optimized	default	optimized
#peaks						
training set:	55,845	57,075	33,298	31,710	24,247	24,230
test set:	65,851	53,205	34,415	32,397	27,539	25,609
#RP^a^						
training set:	6,999	8,433	12,606	14,367	2,710	3,351
test set:	7,587	7,903	12,999	14,594	1,582	1,869
#LIP^b^						
training set:	15,497	11,645	15,245	17,284	11,327	11,490
test set:	11,163	10,855	15,643	17,680	12,646	10,962
PPS^c^						
training set:	1,214	1,565	8,802	14,308	568	881
test set:	1,053	1,475	9,001	14,472	168	238
RCS^d^						
training set:	12.3	144.8	67.8	575.4	92.8	311.8
test set:	9.4	142.4	37.6	580.4	48.1	206.7
#reliable groups						
training set:	536	990	3,669	5,343	1,504	2,424
test set:	314	759	1,564	5,639	793	1,855
#non-reliable groups						
training set:	2,636	82	3,605	151	1,217	101
test set:	2,740	70	3,248	110	1,150	69
GS^e^						
training set:	109	11,952	3,734	189,057	1,859	58,176
test set:	36	8,230	753	289,076	547	49,870

^a^reliable peaks; ^b^low intensity peaks; ^c^peak picking score; ^d^retention time correction; score; ^e^grouping score

### Metabolite fingerprinting in human serum (HILIC method)

**Figure 2:**
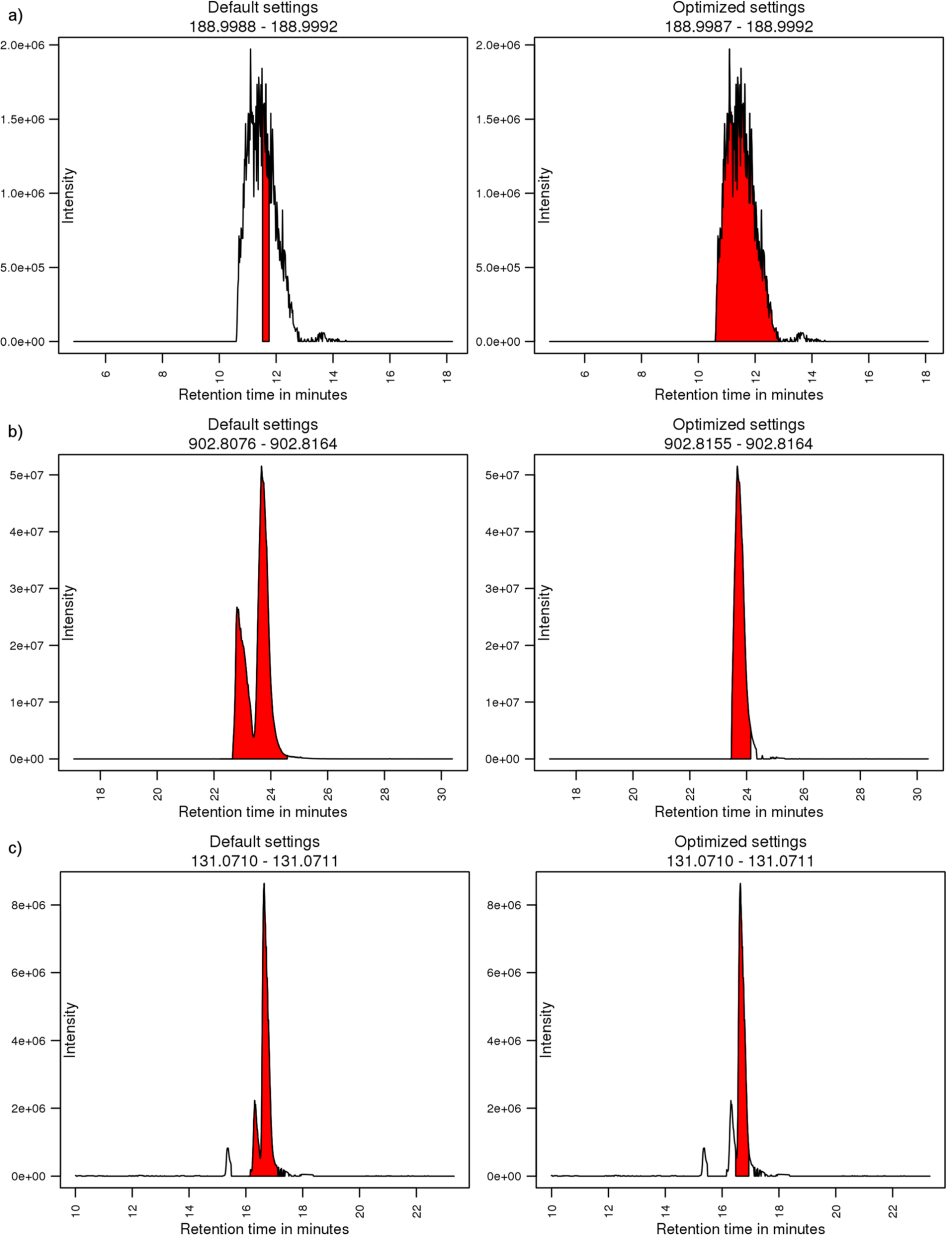
Selected chromatograms showing the different peak types at well-defined masses obtained from the different data sets. Chromatograms derive from **a)** metabolite fingerprinting data set; **b)** lipidomics data set; **c)** central carbon metabolism data set. Peaks derived from default parameters are presented in the left chromatograms and peaks coming from optimized parameters are shown in the chromatograms on the right side, respectively. The peak area integrated by XCMS is colored red. The m/z range for the chromatogram was chosen from the respective minimum and maximum m/z values of the particular peak. Comparison of chromatograms **a)** clearly demonstrate that default peak width parameters were too small for the broad peaks, **b)** shows an example where the mass range used in the default settings was too wide and **c)** illustrate peaks where the default peak width parameters were too wide.

The metabolite fingerprinting data set used hydrophilic interaction chromatography (HILIC) [20] which typically creates broad peaks. Twelve injections of a pooled sample were used as training set for the parameter optimization and eleven different injections were used as test set. All parameters which were not chosen for optimization were kept at their default values. The PPS of the training set increased by 29% from 1,214 to 1,565 and the PPS of the test set increased by 40% from 1,053 to 1,475. Optimization of the peak picking parameters finished after four DoEs and took about four hours. The number of peaks increased from 55,845 to 57,075 in the training set and decreased from 65,851 to 53,205 in the test set. The number of reliable peaks increased from 6,999 to 8,434 in the training set and from 7,587 to 7,903 in the test set. The optimized peak width parameter lay between 32.2 and 95 seconds. Selected chromatograms, showing the different peak types at distinct masses obtained from the different example data sets are shown in Figure 2. The chromatograms in Figure 2a reveal that the default settings for the peak width parameter can be too small. This results in an only partial integration of the peak, whereas the optimized peak width parameter integrates the peak accurately. The optimization of the retention time correction and grouping parameter finished after five DoEs and 0.8 hours. RCS of the training set increased tenfold by using the optimized settings compared to the RCS of the training set calculated with the default parameters. In the test set the increase of RCS was fifteenfold. The number of ‘reliable groups’ increased from 536 to 990, the number of ‘non-reliable groups’ decreased from 2,636 to 82 in the training set. In the test set the number of ‘reliable groups’ increased from 314 to 759 and the number of ‘non-reliable groups’ decreased from 2,740 to 70.

### Lipidomics (RP-HPLC method)

For the lipidomics data set reversed phase high performance liquid chromatography (RP-HPLC) [21] was coupled to a HRMS device. Eight pooled sample injections were analysed. Four of them were used as training set for the optimization process and the four remaining measurements were used as test set. The peak picking parameter ‘noise’ was set to 20,000. All other parameters were kept at their default values. Optimization of peak picking parameters was finished after three DoEs, which took 1.5 hours. Comparing default to optimized settings the amount of peaks decreased from 33,298 to about 31,710 in the training set and from 34,415 to 32,397 in the test set. The number of RPs increased from 12,606 to 14,367 in the training and from 12,999 to 14,594 in the test set. PPS of the test set increased by 61%, from 9,001 to 14,472. The increase of PPS achieved in the training set was 63% from 8,802 to 14,308. The chromatograms in Figure 2b suggest that the default setting for the ‘ppm’ parameter is too large for peaks generated by HRMS. The optimized parameter results in an m/z range of only 1 ppm for the optimized peak, whereas the default peak spans a range of 9.7 ppm. Parameters for retention time correction and grouping needed 0.7 hours and five DoEs to finish. RCS increased more than eightfold in the training set and fifteenfold in the test set. The amount of ‘non-reliable groups’ decreased from 3,605 to 151 in the training set and from 3,248 to 110 in the test set. The number of ‘reliable groups’ increased from 3,669 to 5,343 in the training set and from 1,564 to 5,639 in the test set.

### Central carbon metabolism (IP-RP-HPLC method)

The central carbon metabolism data set utilized a modified ion pair-reversed phase-high performance liquid chromatography IP-RP-HPLC [22] method which exhibits an outstanding separation performance, thereby producing very sharp peaks. All parameters that had not been optimized were kept at their default values. Six injections of a pooled sample were used as training set for parameter optimization and six different injections were used as test set. Optimization of peak picking finished after two DoEs and took 0.9 hours. Within the optimization process, the PPS was increased from 568 achieved with the default parameter settings to 881 in the training set and from 168 to 238 in the test set. The chromatograms in Figure 2c show that default settings for the ‘peakwidth’ parameter are too high for the very sharp peaks generated by this method. The optimization of the retention time correction and grouping parameters for the central carbon metabolism data set finished after four DoEs in 0.6 hours. RCS was more than tripled from 92.8 to 311.8 in the training set and increased fourfold from 48.1 to 206.7 in the test set. ‘Non-reliable groups’ decreased from 1,217 to only 101 and ‘reliable groups’ increased from 1,504 to 2,424 which led to a highly increased GS in the training set. In the test set the ‘non-reliable groups’ decreased from 1,150 to 69 and the ‘reliable groups’ increased from 793 to 1,855.

The total optimization for the metabolite fingerprinting data set took 3.8 hours, the optimization time for the lipidomics data set took 1.5 hours and the optimization of the central carbon metabolism data set needed 0.9 hours. IPO is also intended to be used by inexperienced users. Therefore, all parameters optimized by IPO start at their respective default values and in a fixed range. Experienced users can further reduce the optimization time by starting with settings closer to their expected parameter values. In general, the results showed that IPO successfully optimized peak picking parameters for data from different LC-methods and different kinds of samples. Peaks coming from the IP-RP-HPLC should be the sharpest of all three studies which is confirmed by the peak width statistics (Table 3). Also, observed peak widths for the metabolite fingerprinting and the lipidomics data sets were in good agreement with the expected peak widths for the respective LC-methods. Especially for broader peaks, the optimized parameters showed a much better peak picking performance than the default settings.

**Table 3:** **Peak width parameter settings and resulting peak width statistics of the training sets**

	**Metabolite fingerprinting**		**Lipidomics**		**Central carbon metabolism**	
	**Default**	**Optimized**	**Default**	**Optimized**	**Default**	**Optimized**
‘peakwidth’ parameter [sec]	20-50	32.2-95	20-50	29.6-80	20-50	10-35
mean peak width [sec]	44.2	57.9	44.6	58.4	27.3	15.6
median peak width [sec]	40.6	52.2	41.8	54.5	24.4	12.6
modal peak width [sec]	38.9	51.3	41.4	56.8	10.3	5.8

## Conclusions

We introduced the software package IPO, ‘Isotopologue Parameter Optimization’, performing parameter optimization for the open source R-package XCMS. IPO exploits the existence of natural, stable ^13^C isotopes that are ubiquitous in all biological samples. IPO was applied to LC-HRMS data from tissue, serum and yeast samples and the results showed that it is applicable to data from different types of samples as well as from different LC-MS devices and methods. The optimization time has been remarkable reduced by separating optimization for peak picking parameters from optimization for retention time correction and grouping parameters. IPO is also suitable for XCMS beginners, because the default settings are the start values of the optimization process.

We recommend a powerful workstation with multiple processors and cores, which costs only a fraction of the enormous costs of a modern LC-MS instrument and will enable the user to exploit the full potential of the LC-MS.

IPO is continuously improved, optimization of additional XCMS methods will be implemented, other DoE evaluation techniques will be tested and additional identification of isotopic peaks with the R-package CAMERA [23] will be made available to further increase the power of IPO.

## Availability and requirements

**Project name:** IPO

**Project home page:** https://github.com/glibiseller/IPO

**Operating system(s):** Platform independent

**Programming language:** R

**Other requirements:** xcms, rsm

**License:** GNU GPL

**Any restrictions to use by non-academics:** none

## Additional files

Additional file 1:
**Default levels used in first DoE.** The file shows the default levels used by IPO in the first DoE for the different XCMS methods **(Table S1)**.
Additional file 2:
**Materials.** This file contains a detailed description of the three data sets and information on the computation platform used for optimization.
Additional file 3:
**Response surface models.** This file contains the response surface models of all optimization steps of the three data sets.

## Abbreviations

ARTS: Average retention time shift
BBD: Box-Behnken design
DoE: Design of experiments
GRTS: Group retention time shift
GS: Grouping score
HILIC: Hydrophilic interaction chromatography
HRMS: High resolution mass spectrometry
IA: Isotopic abundance
IP-RP-HPLC: Ion pair-reversed phase-high performance liquid chromatography
LC: Liquid chromatography
LC-MS: Liquid chromatography coupled to mass spectrometry
LIP: Low intensity peaks
PPS: Peak picking score
RCS: Retention time correction score
RGTV: Retention time correction and grouping target value
ROI: Region of interest
RP: Reliable peaks
RP-HPLC: Reversed phase high performance liquid chromatography
