# Supplementary material for: IPO: a tool for automated optimization of XCMS parameters
Source: BMC Bioinformatics. 2015 Apr 16;16:118. doi: 10.1186/s12859-015-0562-8 (PMC4404568; doi:10.1186/s12859-015-0562-8)
Supplement: Additional file 1: — Default levels used in first DoE. The file shows the default levels used by IPO in the first DoE for the different XCMS methods (Table S1). [file 12859_2015_562_MOESM1_ESM.pdf]

Table1: Levels used in first DoE

Parameter	Level 1	Level 2 (center point)	Level 3
**findPeaks.centWave**			
min peakwidth	10	20	30
max peakwidth	35	50	65
ppm	15	25	35
mzdiff	-0.001	0.0055	0.01
snthresh	10		
noise	0		
prefilter	c(3,100)		
mzCenterFun	"wMean"		
integrate	1		
fitgauss	FALSE		
**findPeaks.matchedFilter**			
fwhm	25	30	25
snthresh	3	10	17
step	0.05	0.10	0.15
steps	1	2	3
sigma	fwhm / 2.3548		
max	5		
mzdiff	0.8 - step * steps		
index	FALSE		
**retcor.obiwarp**			
gapInit	0.0	0.2	0.4
gapExtend	2.1	2.4	2.7
profStep	0.7	0.85	1
plotType	"none"		
response	1		
factorDiag	2		
factorGap	1		
localAlignment	0		
initPenalty	0		
**group.density**			
bw	22	30	38
minfrac	0.3	0.5	0.7
mzwid	0.015	0.02	0.035
minsamp	1		
max	50		
