# Supplementary material for: IPO: a tool for automated optimization of XCMS parameters
Source: BMC Bioinformatics. 2015 Apr 16;16:118. doi: 10.1186/s12859-015-0562-8 (PMC4404568; doi:10.1186/s12859-015-0562-8)
Supplement: Additional file 2: — Materials. This file contains a detailed description of the three data sets and information on the computation platform used for optimization. [file 12859_2015_562_MOESM2_ESM.pdf]

Metabolite fingerprinting in human serum (HILIC method)

Serum samples were processed as published by Yuan et al [1]. From all samples, 10 µl were taken and mixed together to generate a pooled sample. LC-MS analyses were performed with an Ultimate 3000 UHPLC system (Thermo Fisher Scientific, San Jose, CA, USA) coupled to a high resolution mass spectrometer Q-Exactive (Thermo Fisher Scientific, Bremen, Germany). Chromatographic separation was achieved on a Luna NH2 column (2×150 mm; 3 µm; Phenomenex, Torrance, USA) following the procedure published by Bajad et al [2]. Full scan spectra were recorded in positive electrospray from m/z 70–1050 with a resolution of 140,000 (m/z 200). After every third sample a pair of a blank and a pooled sample were measured. Twelve measurements were used as training set for parameter optimization and eleven different measurements were used as test set.

Lipidomics (RP-HPLC method)

Tissue samples from muscle, lung and proventriculus of mice were collected and processed, using the method published by Fauland et al [3]. LC-MS analyses were performed with an Ultimate 3000 HPLC system (Thermo Fisher Scientific, San Jose, CA, USA) coupled to a high resolution mass spectrometer Q-Exactive (Thermo Fisher Scientific, Bremen, Germany). Chromatographic separation was achieved on a Hypersil GOLD column (100 mmx1 mm, 1.9 µm; Thermo Fisher Scientific, San Jose, CA, USA). Full scan spectra were recorded from m/z 350–1,050 with a resolution of 140,000 (@ m/z 200) using heated positive electrospray ionization. After every sixth sample a pair of a blank and a pooled sample were measured. The measurements of the eight injections

of the pooled sample were split into a training set and a test set with four measurements each.

Central carbon metabolism (IP-RP-HPLC method)

Metabolites were extracted from stationary phase yeast cells (BY4741 background strain). Culture aliquots of OD_{600} were harvested by filtration with 0.22 µm sterile filters, washed once (on filter) with 5 ml double-distilled water and were immediately quenched by deep-freezing the filters in liquid nitrogen. The filtration and the washing step were performed in less than 30 seconds before the freezing step. For the acid extraction of metabolites, cells were resuspended in 1 ml ice-cold 5% trichloroacetic acid (TCA) and incubated for 1 hour on ice with occasional vortexing. Supernatants (10 min; 10,000 g) were lyophilized and resuspended in 200 µl double-distilled water. Aliquots of each cell extract were pooled.

LC-MS analyses were performed with an Ultimate 3000 HPLC system (Thermo Fisher Scientific, San Jose, CA, USA) coupled to a high resolution mass spectrometer Exactive™ Orbitrap system. Chromatographic separation was achieved on an Atlantis T3 C18 analytical column (150 mm x 3 mm, 3 µm, Waters, USA). HPLC was run with a two eluent multi-step gradient of 2-propanol and an aqueous mobile phase (5 % methanol (*v/v*), 10 mM tributylamine (TBA) and 15 mM acetic acid, pH 4.95) within 40 minutes per sample [4].

Heated electrospray ionization was used for negative ionization. Data acquisition was conducted via full scan of all masses between 70 and 1,100 m/z (R = 50,000). The injection volume was set to 10 µl per sample. A blank sample and a pooled sample were

measured periodically after every fifth sample. Six pooled sample injections were used as training set, six other injections as test set.

Computational platform

LC-MS raw files of the metabolite fingerprinting and lipidomics data sets were converted to mzXML using ReadW 4.0.2. The files originating from the central carbon metabolism data set were converted using MsConvert of the ProteoWizard release 3.0.5033. Optimization was done on an Intel(R) Core™ i5 CPU 760 @ 2.80GHz system with 4 GB RAM running Windows 7 32 Bit with R (v3.1.1) and using the R-packages XCMS (v.1.40.0), rsm (v.2.07) as well as IPO (v.1.5.5). For optimization of all example data sets four cores were used.

1. Yuan M, Breitkopf SB, Yang X, Asara JM: **A positive/negative ion-switching, targeted mass spectrometry-based metabolomics platform for bodily fluids, cells, and fresh and fixed tissue.** *Nat Protoc* 2012, **7**:872–881.

2. Bajad SU, Lu W, Kimball EH, Yuan J, Peterson C, Rabinowitz JD: **Separation and quantitation of water soluble cellular metabolites by hydrophilic interaction chromatography-tandem mass spectrometry**. *J Chromatogr A* 2006, **1125**:76–88.

3. Fauland A, Köfeler H, Trötzmüller M, Knopf A, Hartler J, Eberl A, Chitraju C, Lankmayr E, Spener F: **A comprehensive method for lipid profiling by liquid chromatography-ion cyclotron resonance mass spectrometry.** *J Lipid Res* 2011, **52**:2314–2322.

4. Buescher JM, Moco S, Sauer U, Zamboni N: **Ultrahigh performance liquid chromatography-tandem mass spectrometry method for fast and robust quantification of anionic and aromatic metabolites.** *Anal Chem* 2010, **82**:4403–4412.
