# Supplementary material for: IPO: a tool for automated optimization of XCMS parameters
Source: BMC Bioinformatics. 2015 Apr 16;16:118. doi: 10.1186/s12859-015-0562-8 (PMC4404568; doi:10.1186/s12859-015-0562-8)
Supplement: Additional file 3: — Response surface models. This file contains the response surface models of all optimization steps of the three data sets. [file 12859_2015_562_MOESM3_ESM.pdf]

Response Surface Models

1. Metabolite fingerprinting in human serum (HILIC method) data set

- Download and install IPO (https://github.com/glibiseller/IPO)
- Download and extract files (https://health.joanneum.at/IPO/MetaboliteFingerprintingTrainingSet.zip)
- Set the working directory to the location of the extracted files: setwd("path_to_extracted_files")
- Start R console and use the following script:
  library(IPO)
  ppParams <- getDefaultXcmsSetStartingParams()
  ppResult <- optimizeXcmsSet(params=ppParams, nSlaves=4)
  rgResult <- optimizeRetGroup(xset=ppResult$best_settings$xset, nSlaves=4, subdir=subdir)
  ppResult$best_settings$parameters
  rgResult$best_settings

- The response surface models will be stored in the subfolder "IPO"

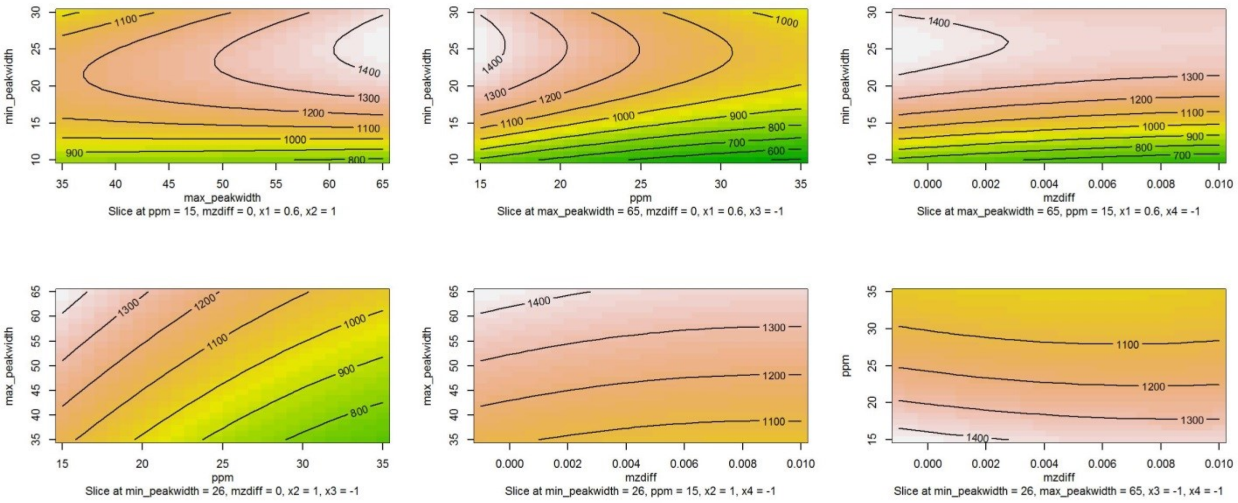

Figure 1-1: Response Surface Models of the first DoE for optimization of peak picking parameters

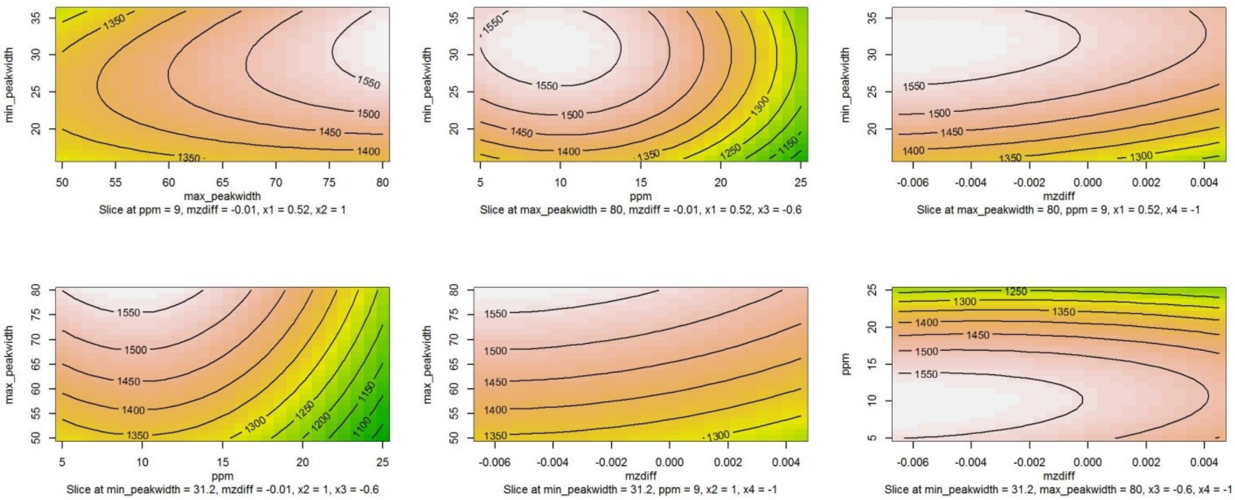

Figure 1-2: Response Surface Models of the second DoE for optimization of peak picking parameters

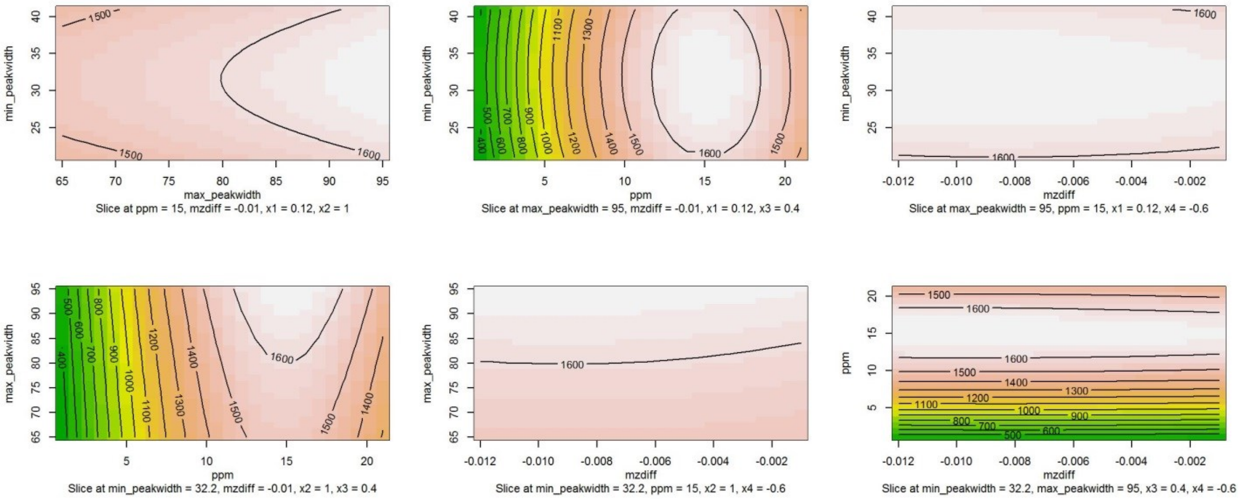

Figure 1-3: Response Surface Models of the third DoE for optimization of peak picking parameters

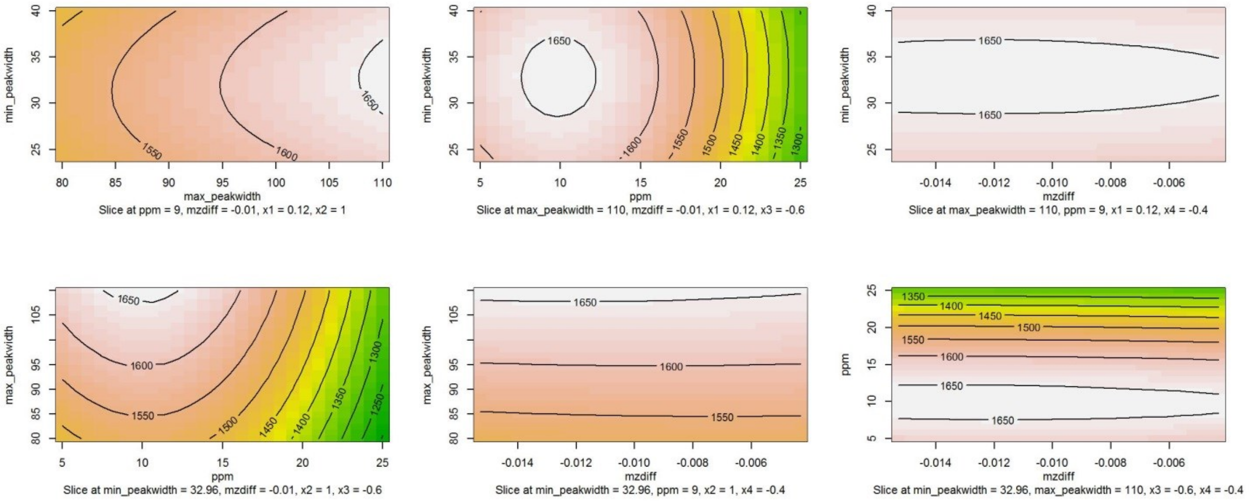

Figure 1-4: Response Surface Models of the fourth DoE for optimization of peak picking parameters

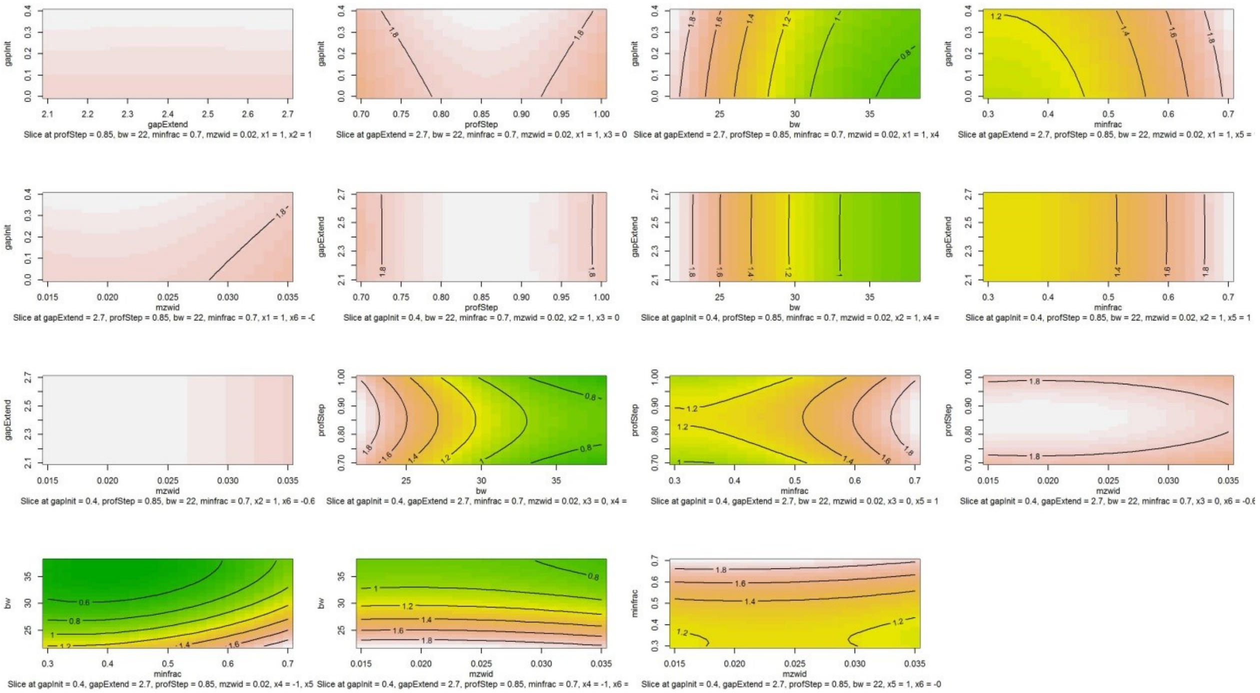

Figure 1-5: Response Surface Models of the first DoE for optimization of retention time correction and grouping parameters

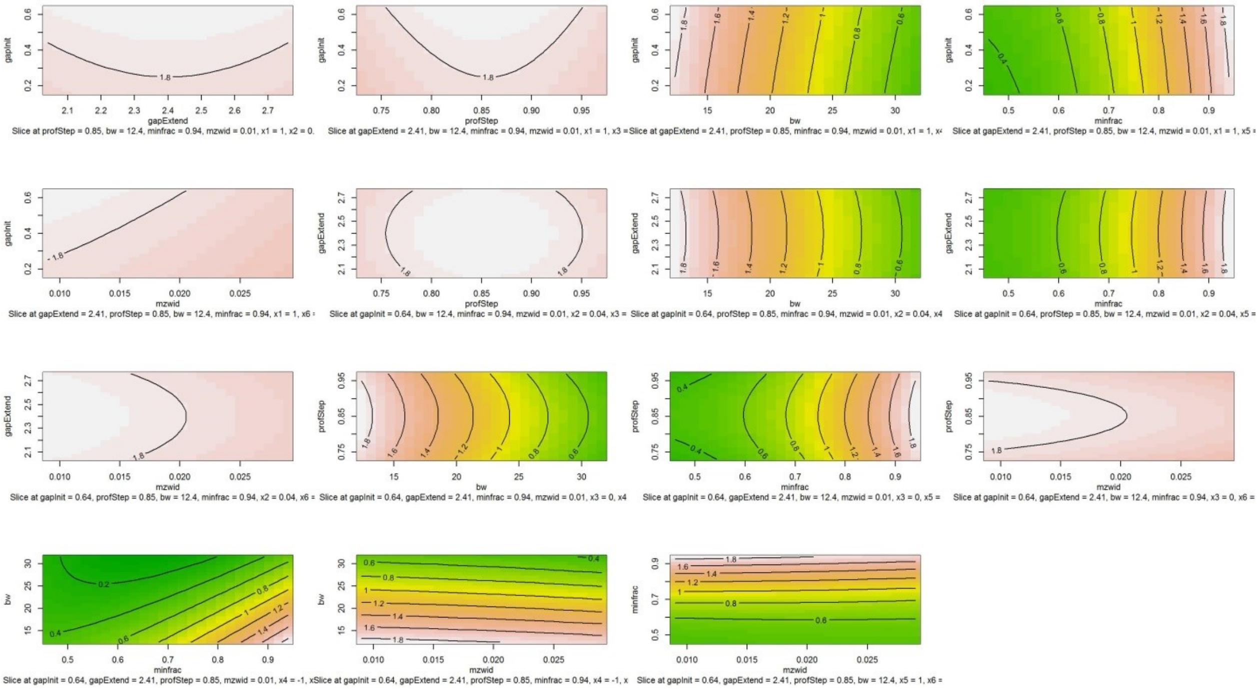

Figure 1-6: Response Surface Models of the second DoE for optimization of retention time correction and grouping parameters

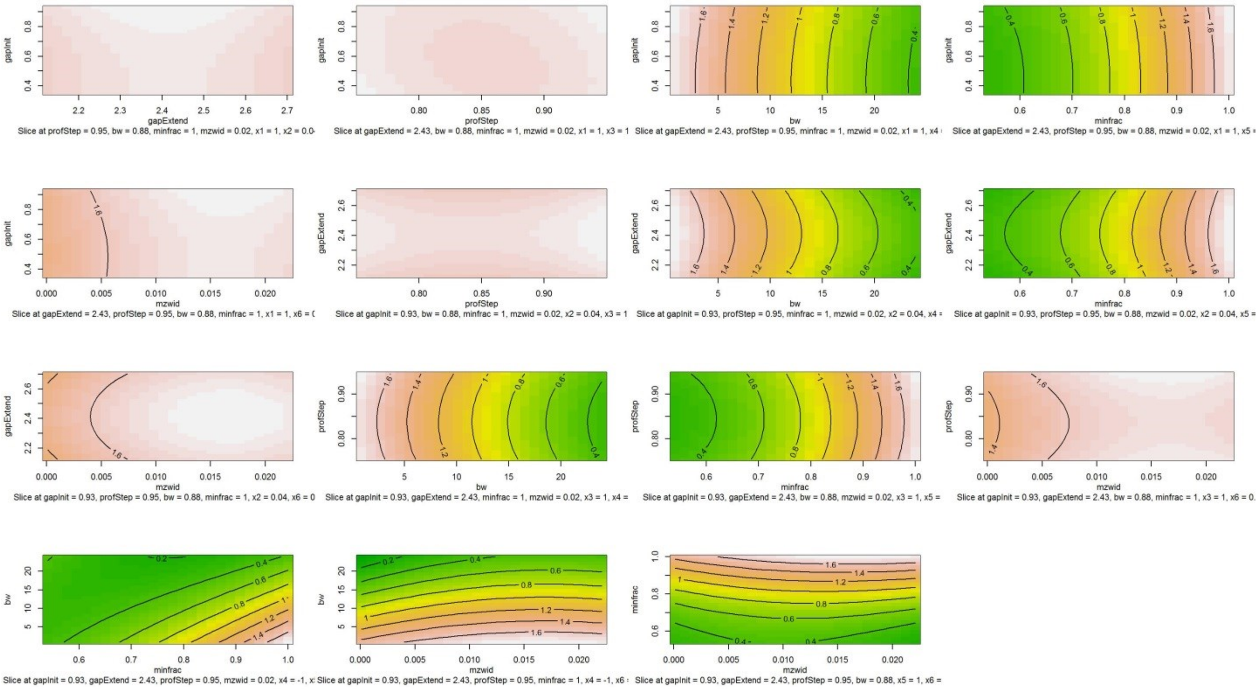

Figure 1-7: Response Surface Models of the third DoE for optimization of retention time correction and grouping parameters

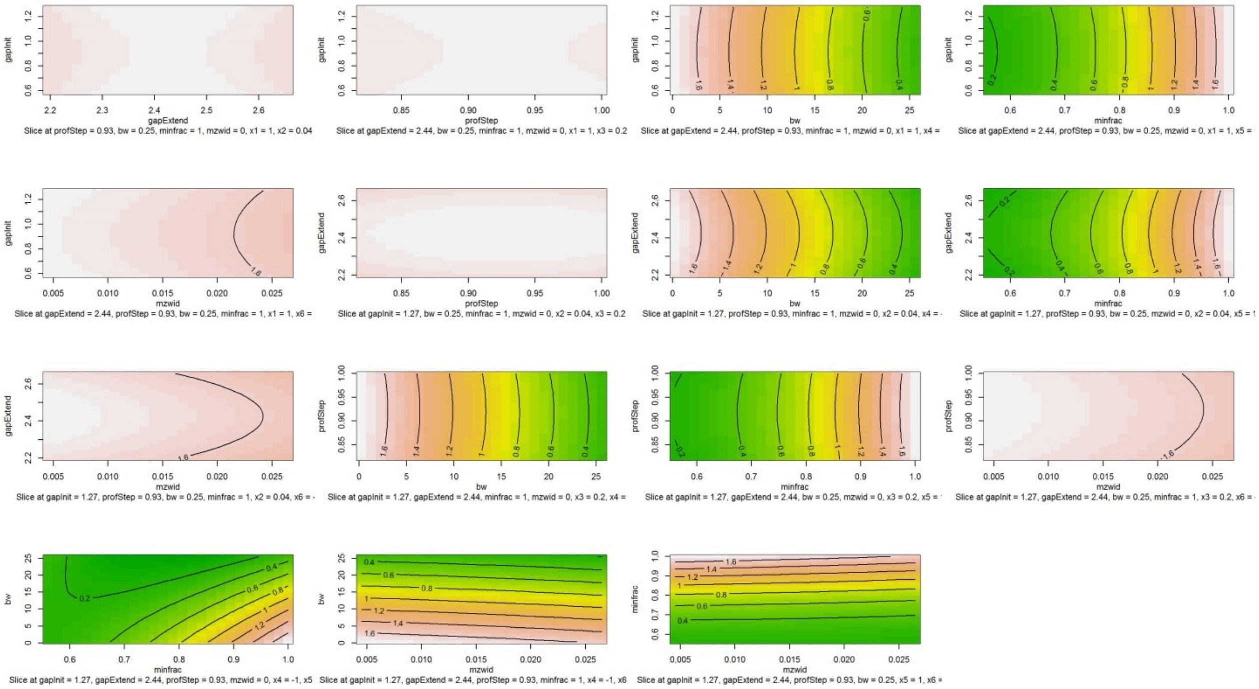

Figure 1-8: Response Surface Models of the fourth DoE for optimization of retention time correction and grouping parameters

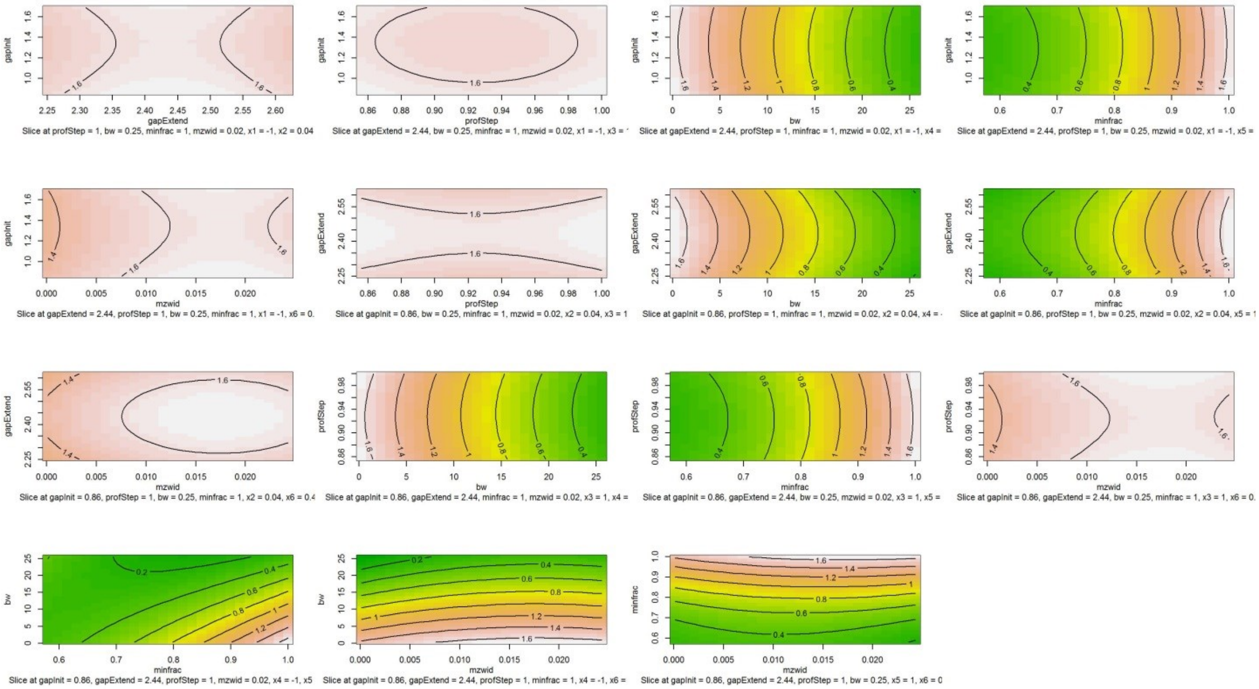

Figure 1-9: Response Surface Models of the fifth DoE for optimization of retention time correction and grouping parameters

2. Lipidomics (RP-HPLC method) data set

- Download and install IPO (https://github.com/glibiseller/IPO)
- Download and extract files (https://health.joanneum.at/IPO/LipidomicsTrainingSet.zip)
- Set the working directory to the location of the extracted files:
  setwd(“path_to_extracted_files”)
- Start R console and use the following script:
  library(IPO)
  ppParams <- getDefaultXcmsSetStartingParams()
  ppParams$noise <- 20000
  ppResult <- optimizeXcmsSet(params=ppParams, nSlaves=4)
  rgResult <- optimizeRetGroup(xset=ppResult$best_settings$xset, nSlaves=4, subdir=subdir)
  ppResult$best_settings$parameters
  rgResult$best_settings

- The response surface models will be stored in the subfolder “IPO”

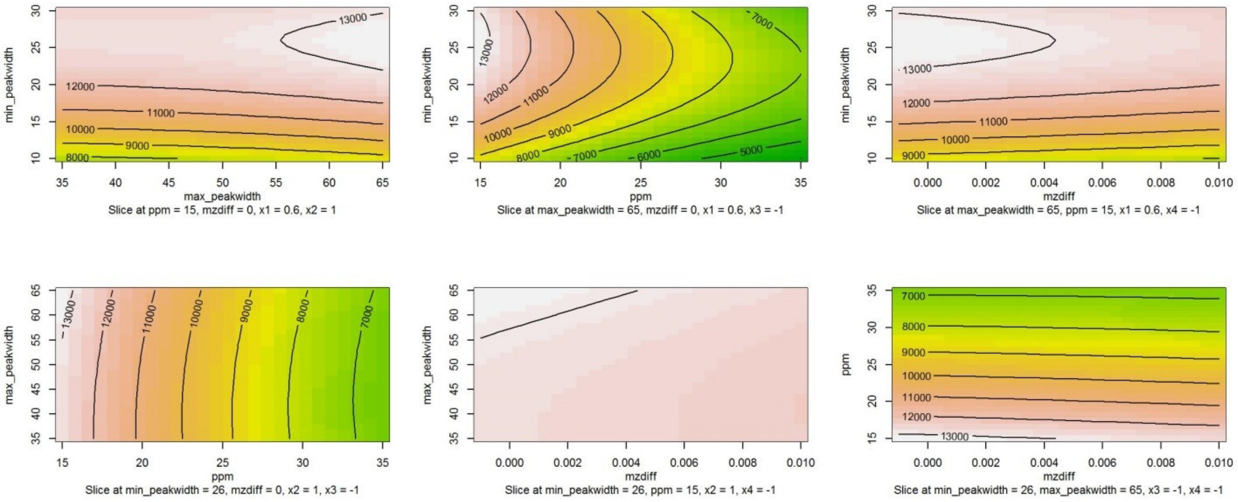

Figure 2-1: Response Surface Models of the first DoE for optimization of peak picking parameters

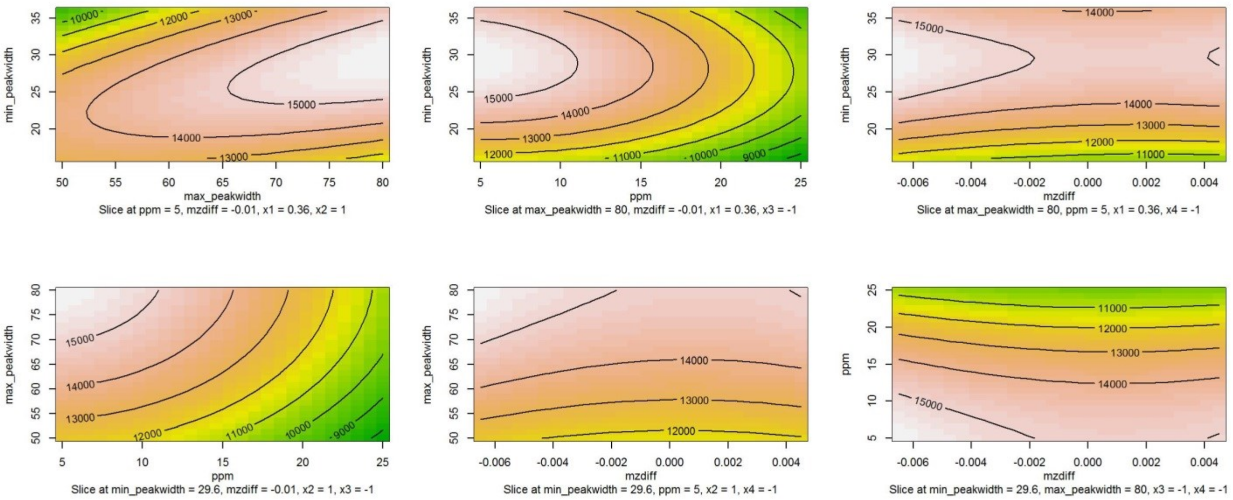

Figure 2-2: Response Surface Models of the second DoE for optimization of peak picking parameters

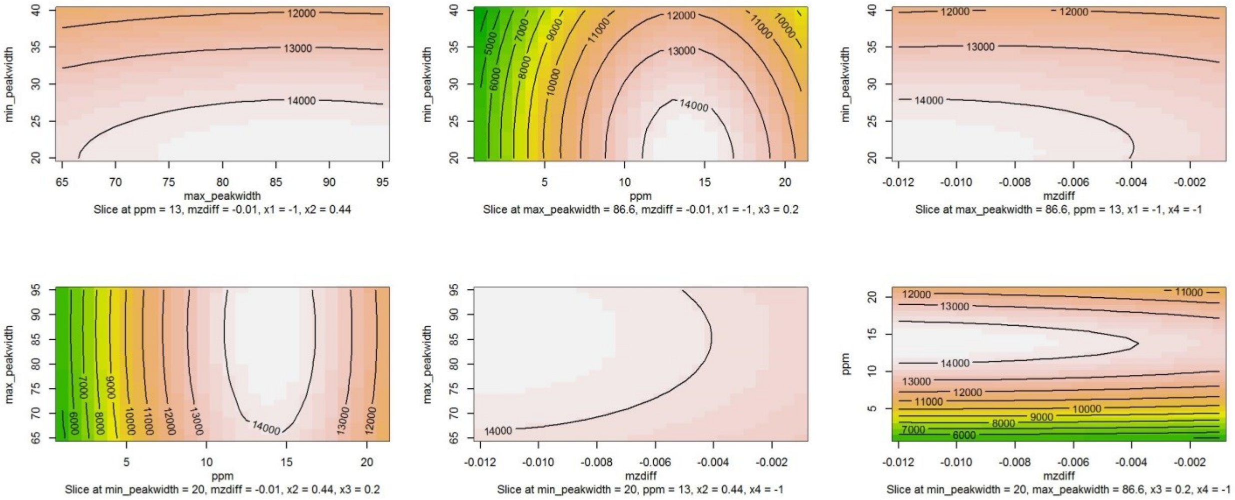

Figure 2-3: Response Surface Models of the third DoE for optimization of peak picking parameters

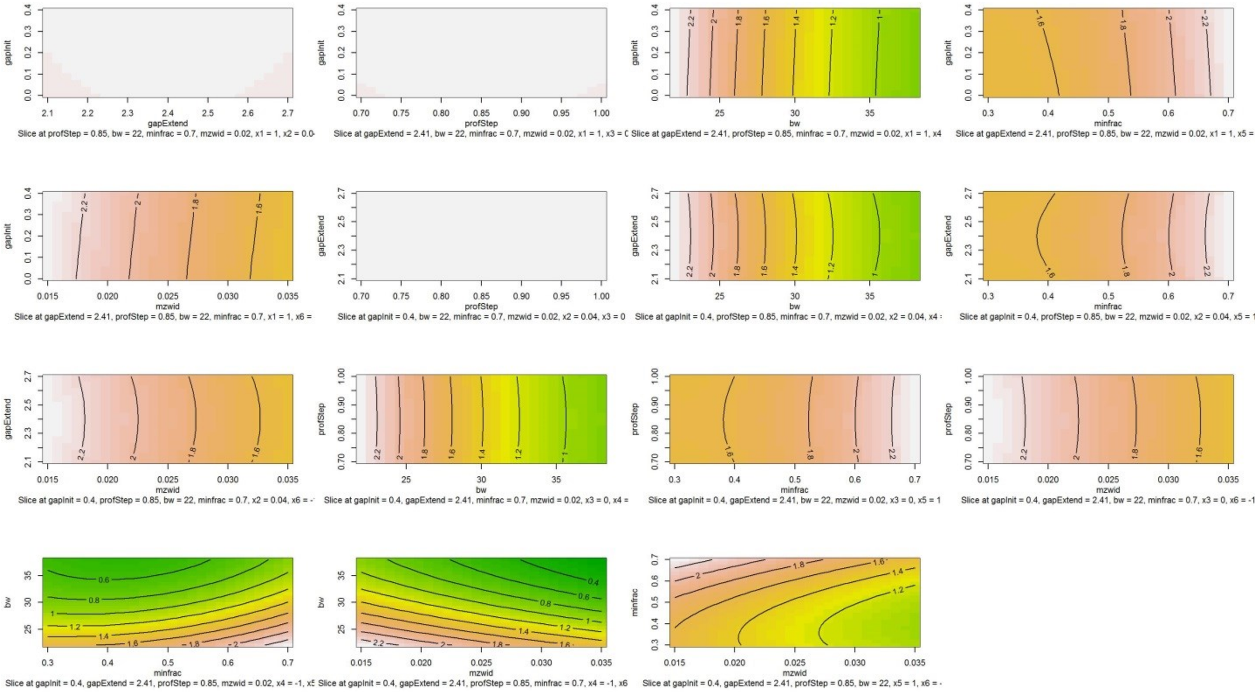

Figure 2-4: Response Surface Models of the first DoE for optimization of retention time correction and grouping parameters

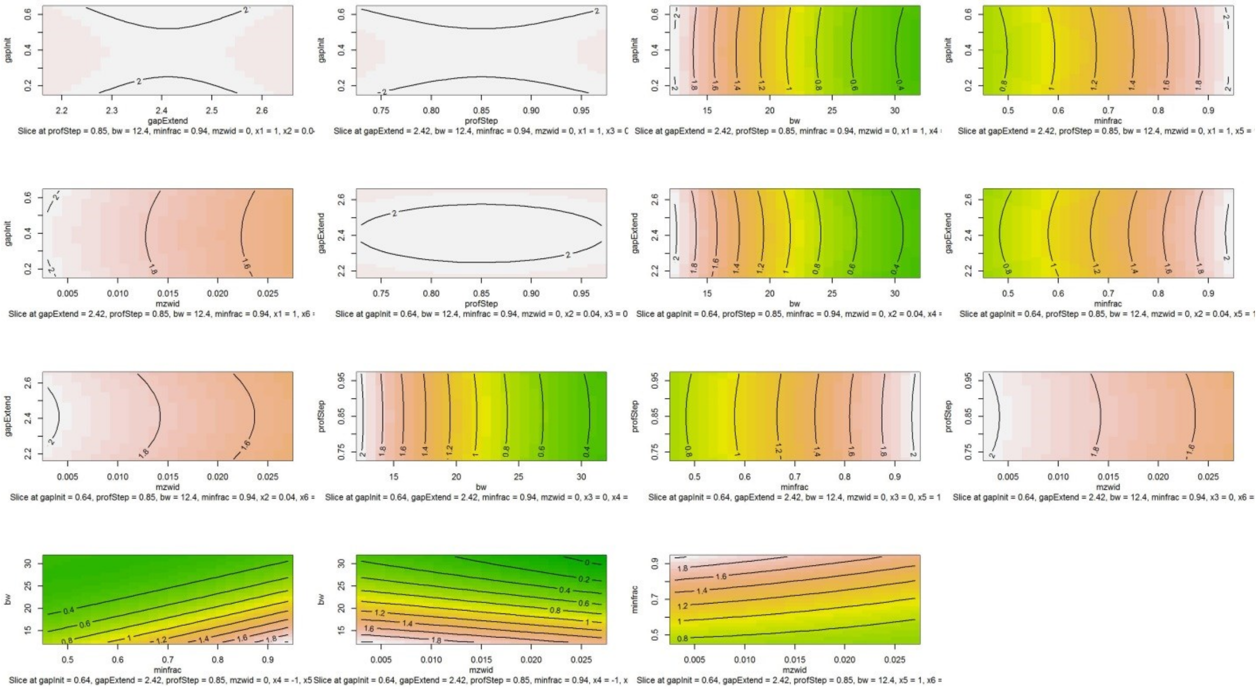

Figure 2-5: Response Surface Models of the second DoE for optimization of retention time correction and grouping parameters

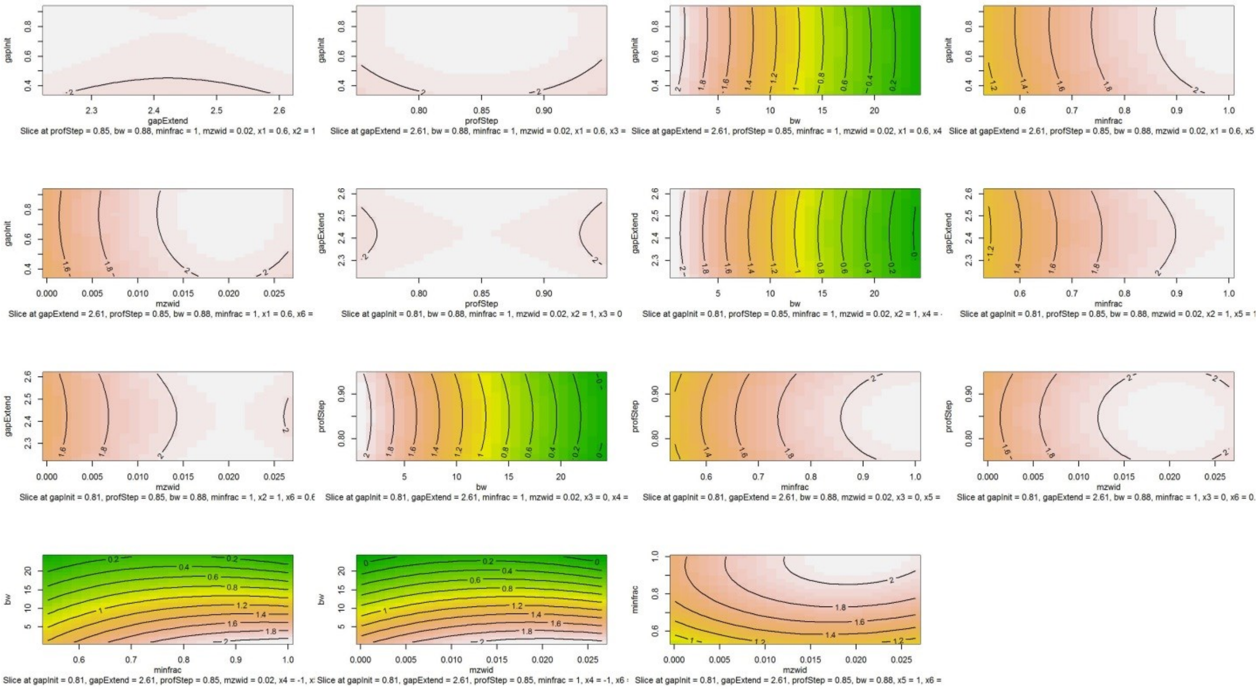

Figure 2-6: Response Surface Models of the third DoE for optimization of retention time correction and grouping parameters

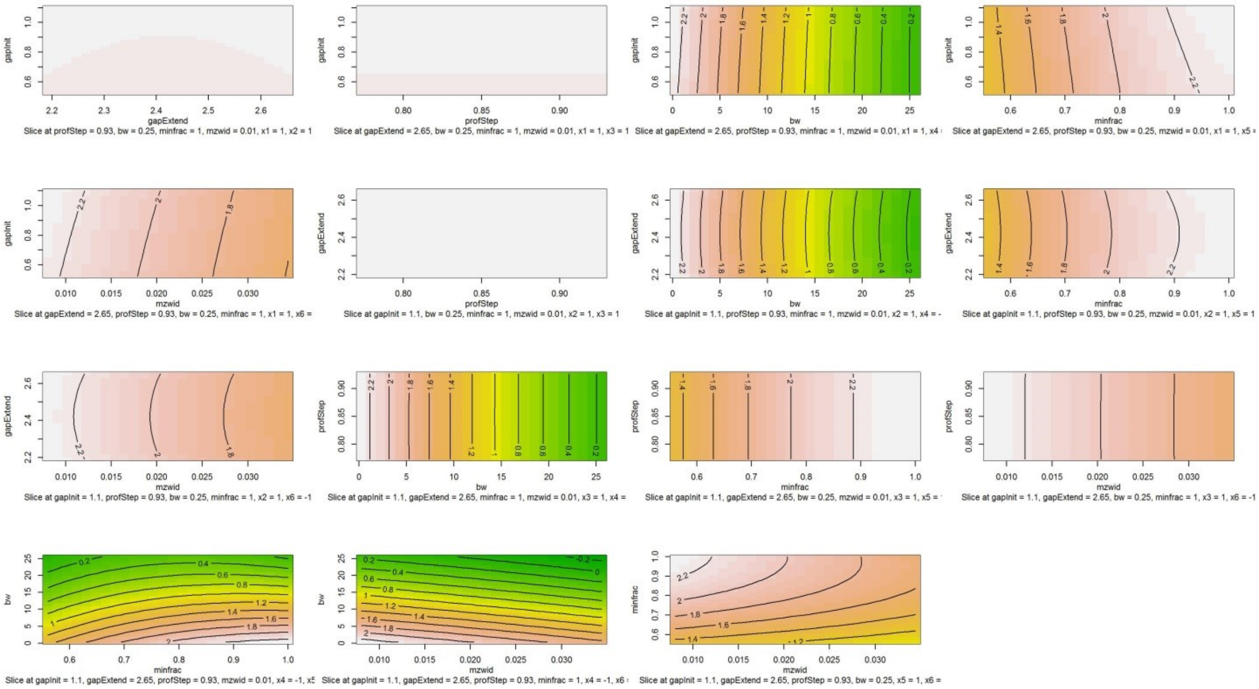

Figure 2-7: Response Surface Models of the fourth DoE for optimization of retention time correction and grouping parameters

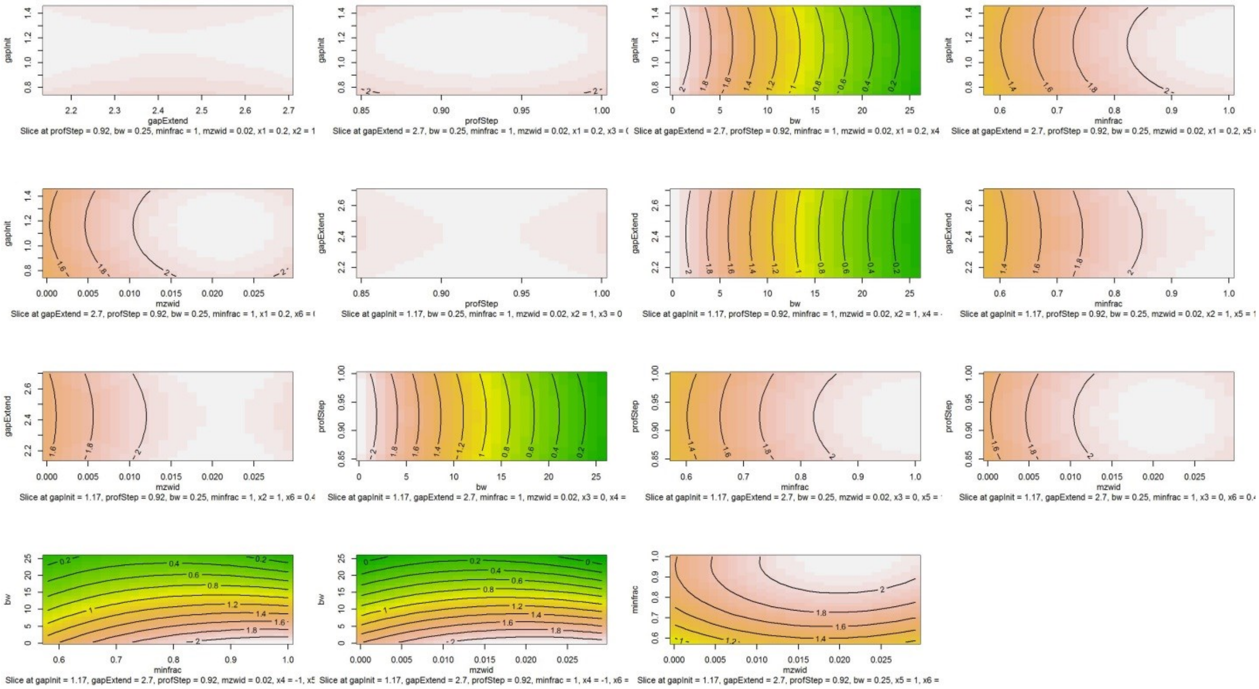

Figure 2-8: Response Surface Models of the fifth DoE for optimization of retention time correction and grouping parameters

3. Central carbon metabolism (IP-RP-HPLC method) data set

- Download and install IPO (https://github.com/glibiseller/IPO)
- Download and extract files (https://health.joanneum.at/IPO/CentralCarbonMetabolismTrainingSet.zip)
- Set the working directory to the location of the extracted files: setwd(“path_to_extracted_files”)
- Start R console and use the following script:

```
library(IPO)
ppParams <- getDefaultXcmsSetStartingParams()
ppResult <- optimizeXcmsSet(params=ppParams, nSlaves=4)
rgResult <- optimizeRetGroup(xset=ppResult$best_settings$xset, nSlaves=4, subdir=subdir)
ppResult$best_settings$parameters
rgResult$best_settings
```

- The response surface models will be stored in a subfolder IPO

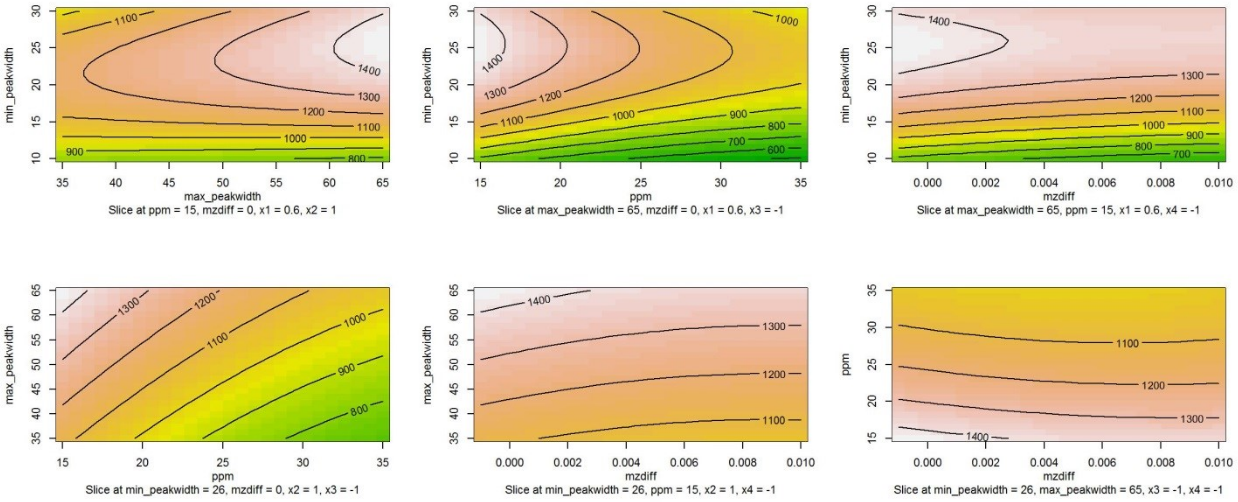

Figure 3-1: Response Surface Models of the first DoE for optimization of peak picking parameters

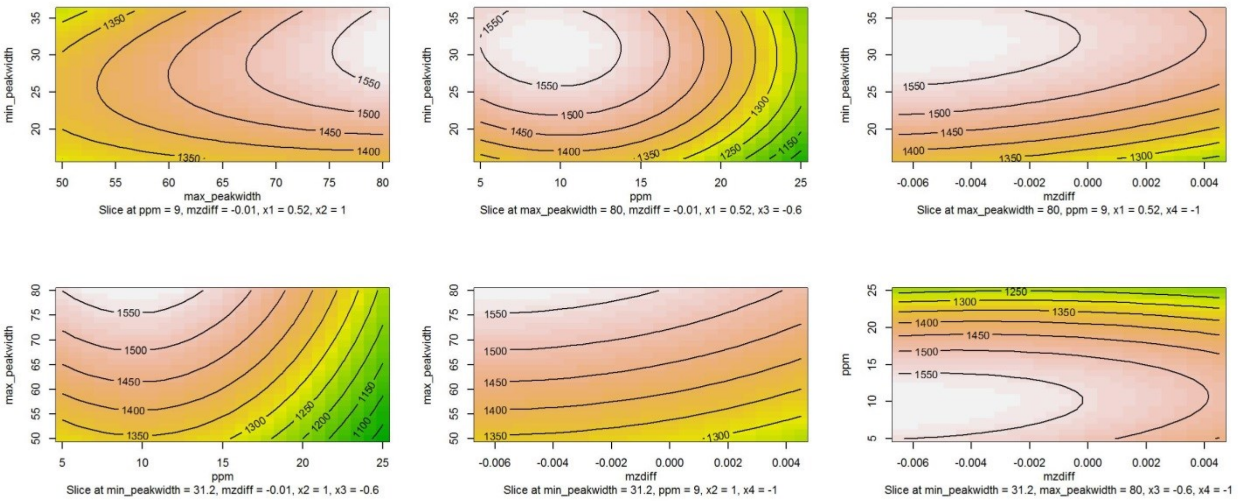

Figure 3-2: Response Surface Models of the second DoE for optimization of peak picking parameters

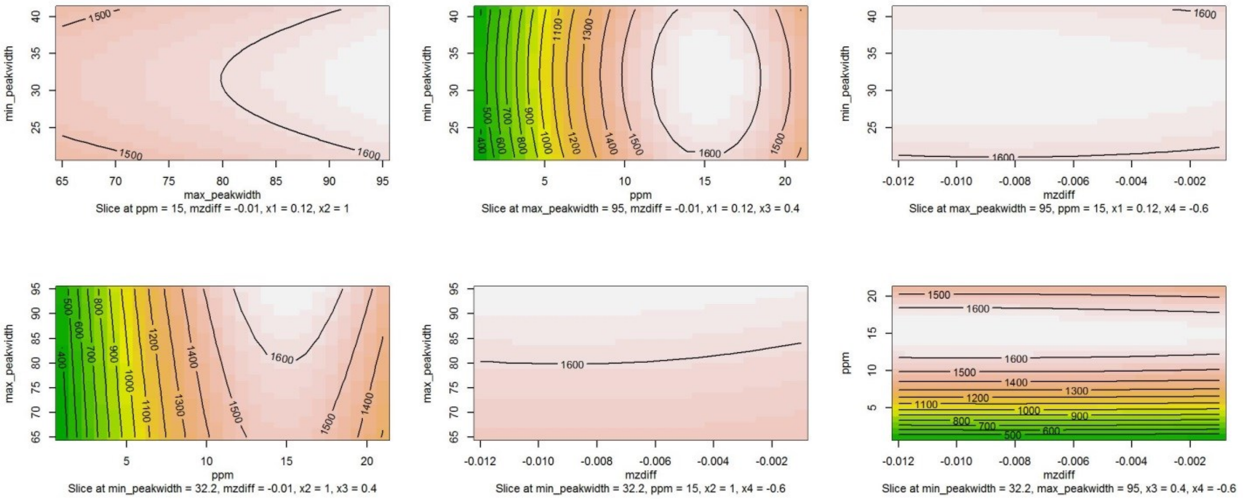

Figure 3-3: Response Surface Models of the third DoE for optimization of peak picking parameters

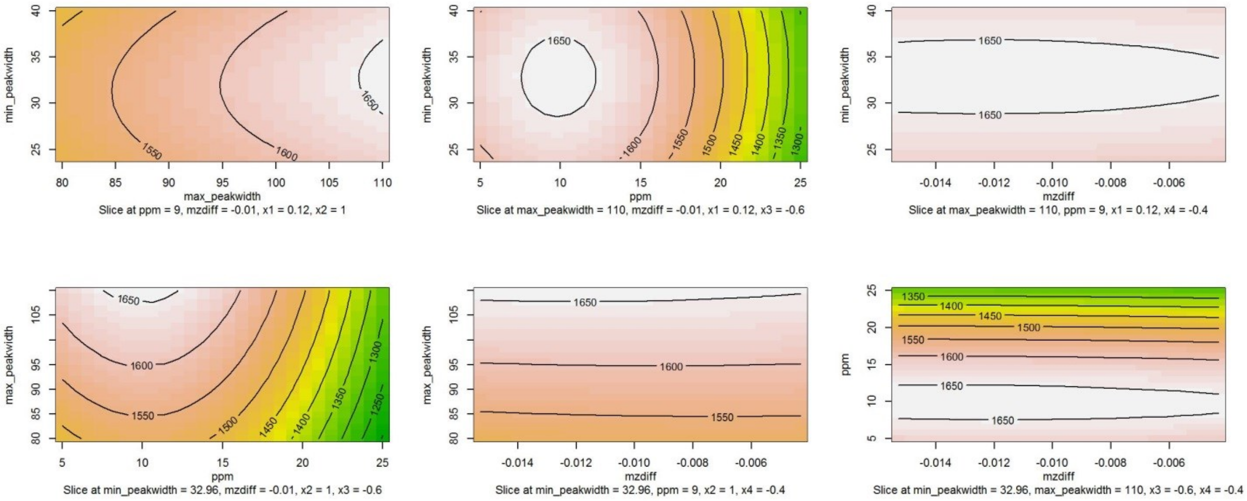

Figure 3-4: Response Surface Models of the fourth DoE for optimization of peak picking parameters

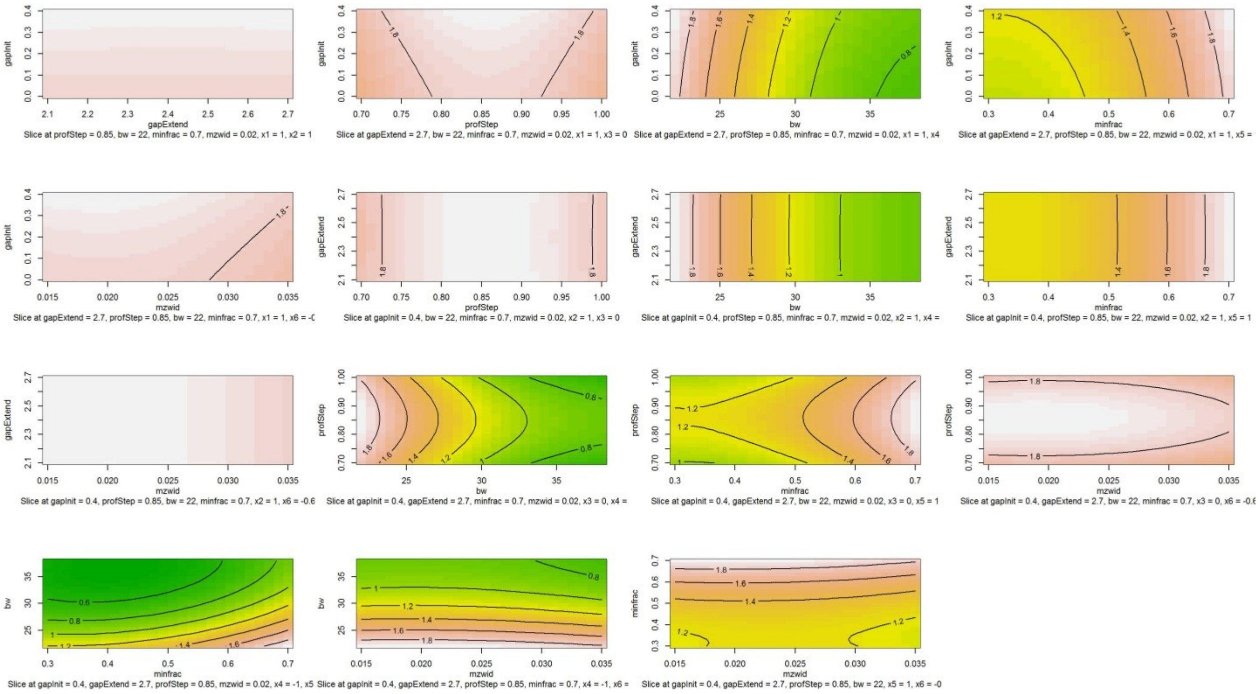

Figure 3-5: Response Surface Models of the first DoE for optimization of retention time correction and grouping parameters

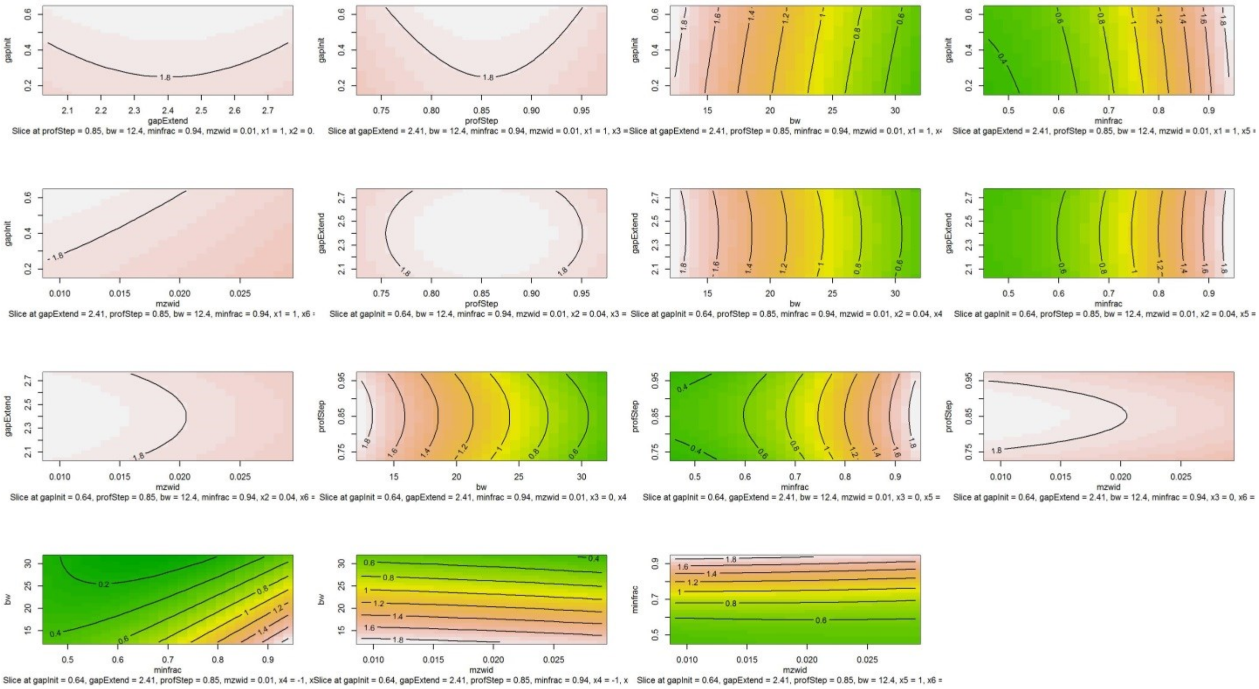

Figure 3-6: Response Surface Models of the second DoE for optimization of retention time correction and grouping parameters

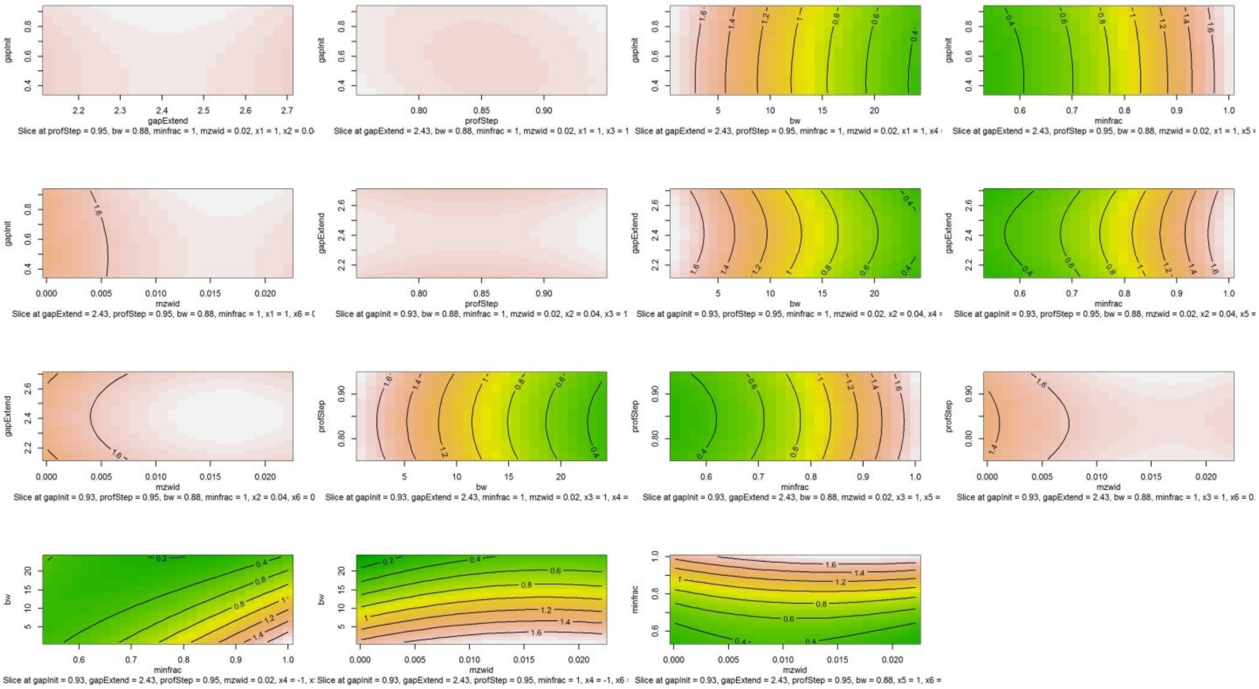

Figure 3-7: Response Surface Models of the third DoE for optimization of retention time correction and grouping parameters

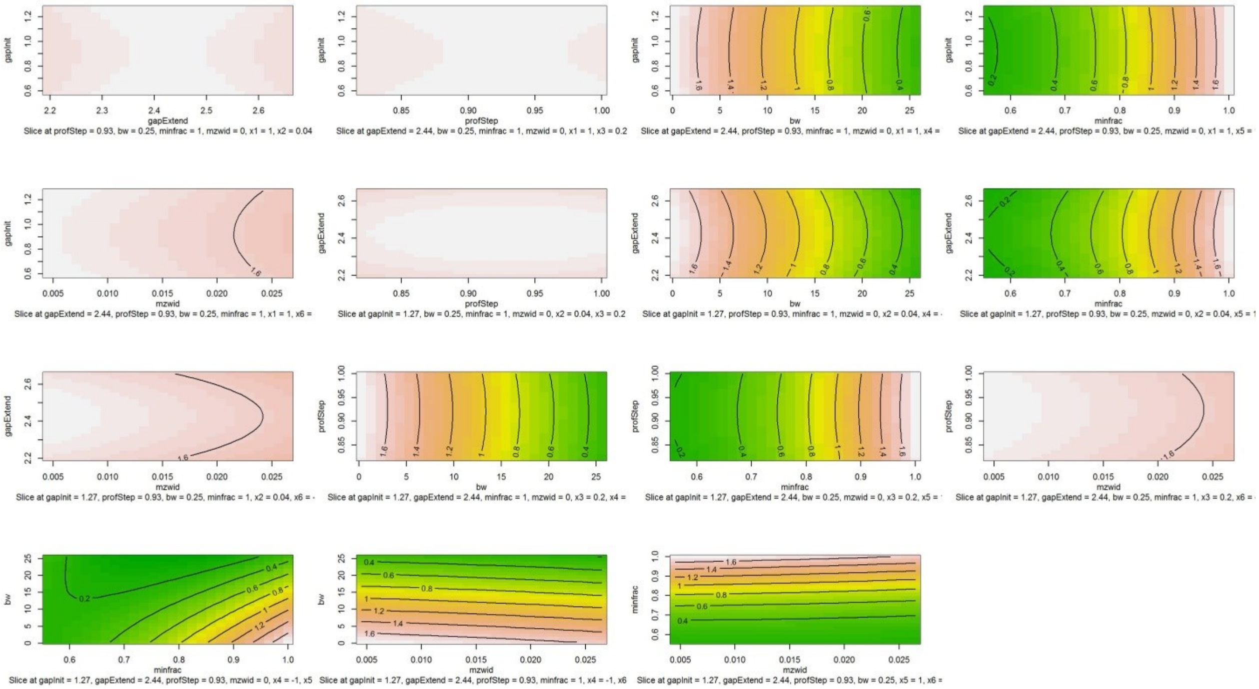

Figure 3-8: Response Surface Models of the fourth DoE for optimization of retention time correction and grouping parameters

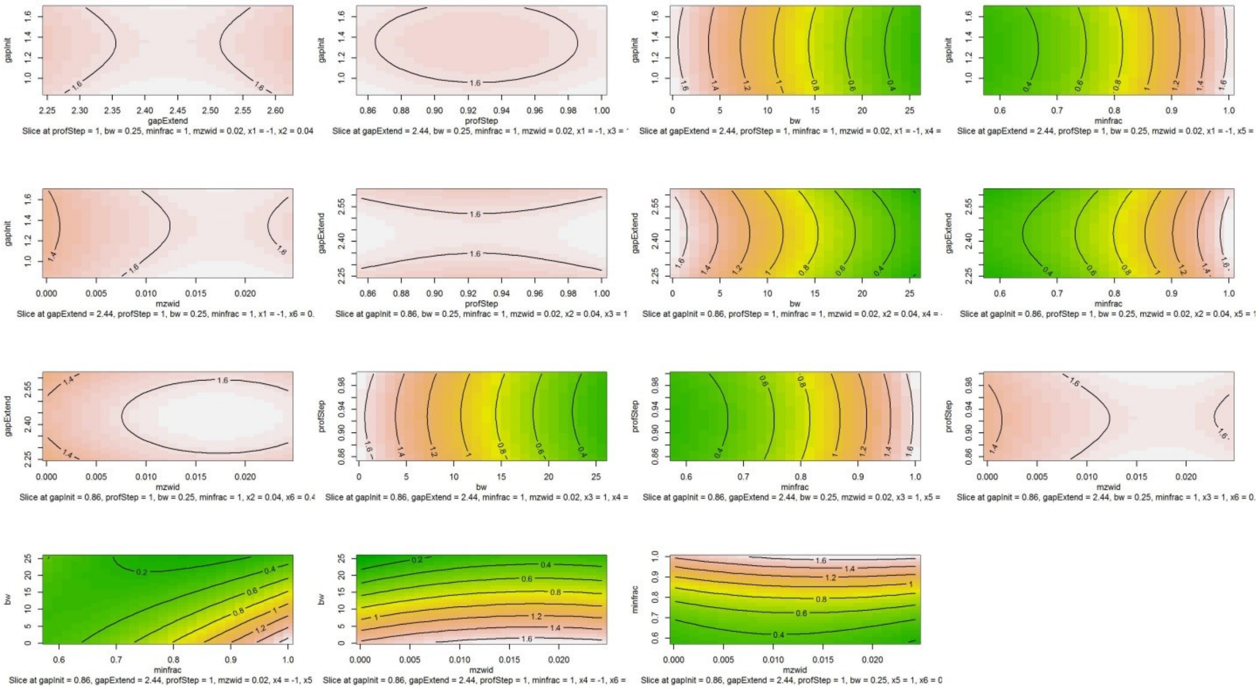

Figure 3-9: Response Surface Models of the fifth DoE for optimization of retention time correction and grouping parameters
